# Admixture in Latin America: Geographic Structure, Phenotypic Diversity and Self-Perception of Ancestry Based on 7,342 Individuals

**DOI:** 10.1371/journal.pgen.1004572

**Published:** 2014-09-25

**Authors:** Andrés Ruiz-Linares, Kaustubh Adhikari, Victor Acuña-Alonzo, Mirsha Quinto-Sanchez, Claudia Jaramillo, William Arias, Macarena Fuentes, María Pizarro, Paola Everardo, Francisco de Avila, Jorge Gómez-Valdés, Paola León-Mimila, Tábita Hunemeier, Virginia Ramallo, Caio C. Silva de Cerqueira, Mari-Wyn Burley, Esra Konca, Marcelo Zagonel de Oliveira, Mauricio Roberto Veronez, Marta Rubio-Codina, Orazio Attanasio, Sahra Gibbon, Nicolas Ray, Carla Gallo, Giovanni Poletti, Javier Rosique, Lavinia Schuler-Faccini, Francisco M. Salzano, Maria-Cátira Bortolini, Samuel Canizales-Quinteros, Francisco Rothhammer, Gabriel Bedoya, David Balding, Rolando Gonzalez-José

**Affiliations:** 1 Department of Genetics, Evolution and Environment, and UCL Genetics Institute, University College London, London, United Kingdom; 2 National Institute of Anthropology and History, México City, México; 3 Centro Nacional Patagónico, CONICET, Puerto Madryn, Argentina; 4 Universidad de Antioquia, Medellín, Colombia; 5 Instituto de Alta Investigación Universidad de Tarapacá, Programa de Genética Humana ICBM Facultad de Medicina Universidad de Chile and Centro de Investigaciones del Hombre en el Desierto, Arica, Chile; 6 Facultad de Medicina and Facultad de Química, UNAM, México City, México; 7 Departamento de Genética, Universidade Federal do Rio Grande do Sul, Porto Alegre, Brasil; 8 Remote Sensing and Digital Imaging Laboratory, Graduate Program on Geology, Vale do Rio dos Sinos University, São Leopoldo, Brazil; 9 The Institute for Fiscal Studies, London, United Kingdom; 10 Department of Economics, University College London, United Kingdom; 11 Department of Anthropology, University College London, London, United Kingdom; 12 Institute for Environmental Sciences, University of Geneva, Carouge, Switzerland; 13 Laboratorios de Investigación y Desarrollo, Facultad de Ciencias y Filosofía, Universidad Perúana Cayetano Heredia, Lima, Perú; 14 Departamento de Antropología. Facultad de Ciencias Sociales y Humanas. Universidad de Antioquia, Medellín, Colombia; 15 Instituto Nacional de Ciencias Médicas y Nutrición “Salvador Zubirán”, México City, México; University of Chicago, United States of America

## Abstract

The current genetic makeup of Latin America has been shaped by a history of extensive admixture between Africans, Europeans and Native Americans, a process taking place within the context of extensive geographic and social stratification. We estimated individual ancestry proportions in a sample of 7,342 subjects ascertained in five countries (Brazil, Chile, Colombia, México and Perú). These individuals were also characterized for a range of physical appearance traits and for self-perception of ancestry. The geographic distribution of admixture proportions in this sample reveals extensive population structure, illustrating the continuing impact of demographic history on the genetic diversity of Latin America. Significant ancestry effects were detected for most phenotypes studied. However, ancestry generally explains only a modest proportion of total phenotypic variation. Genetically estimated and self-perceived ancestry correlate significantly, but certain physical attributes have a strong impact on self-perception and bias self-perception of ancestry relative to genetically estimated ancestry.

## Author Summary

Latin America has a history of extensive mixing between Native Americans and people arriving from Europe and Africa. As a result, individuals in the region have a highly heterogeneous genetic background and show great variation in physical appearance. Latin America offers an excellent opportunity to examine the genetic basis of the differentiation in physical appearance between Africans, Europeans and Native Americans. The region is also an advantageous setting in which to examine the interplay of genetic, physical and social factors in relation to ethnic/racial self-perception. Here we present the most extensive analysis of genetic ancestry, physical diversity and self-perception of ancestry yet conducted in Latin America. We find significant geographic variation in ancestry across the region, this variation being consistent with demographic history and census information. We show that genetic ancestry impacts many aspects of physical appearance. We observe that self-perception is highly influenced by physical appearance, and that variation in physical appearance biases self-perception of ancestry relative to genetically estimated ancestry.

## Introduction

Understanding the basis of a variation in human physical appearance has been a topic of long-standing research interest. However, little is known about the genetic basis of most of this variation. An exception is pigmentation, which has been the focus of considerable research, particularly in Europeans [1]–[4]. Refining our knowledge on the genetics of physical appearance in human populations is of considerable evolutionary, biomedical and forensic importance. This research is also of broad social interest due to its bearing on debates around notions of self-identity, ethnicity and race.

Latin America provides an advantageous setting in which to examine the impact of genetic variation on physical appearance. The region has a history of extensive admixture between three continental populations: Africans, Europeans and Native Americans [5], [6]. Latin America also provides an informative context in which to explore the perception of variation in physical appearance. The region has a unique history relating to the social and cultural politics of ethnicity, race and nation [7]–[9]. A considerable number of genetic studies have examined admixture in Latin America [10]–[14]. However, these analyses have mostly been based on relatively small samples and focused mainly on describing patterns of variation in admixture proportions between individuals and countries/regions. Few studies have examined the impact of genetic ancestry on physical appearance or the relationship of these to individual notions of ethnicity and ancestry [15], [16].

In this paper we present the first phase of a research program focused on the genetics of physical appearance in Latin Americans. We base this program on a sample of over 7,000 individuals ascertained in five countries: Brazil, Chile, Colombia, México and Perú. Information was obtained for a range of socio-demographic variables, physical attributes and self-perception of ancestry. Here we report analyses based on individual mean genome admixture proportions. Coordinate-based spatial analyses illustrate the significant variation in ancestry existing across Latin America, in agreement with demographic history and census information. Significant effects of ancestry were detected for most of the phenotypes examined, and the direction of these effects agrees with the phenotypic differentiation of Africans, Europeans and Native Americans. Finally, we observe that certain phenotypes have a strong impact on self-perception and that these phenotypes bias self-perceived relative to genetically estimated ancestry.

## Results

Summary descriptive statistics for the study sample collected are presented in Table 1.

**Table 1:** Sample size, proportion of women, age, estimated admixture proportions and phenotypic features of the study sample.

	Brazil	Chile	Colombia	México	Perú	Total
N	1,594	1,561	1,659	1,622	906	7,342
Women	0.67	0.34	0.56	0.61	0.60	0.55
Age	25	24	23	25	20	23
American Ancestry	0.09	0.48	0.29	0.56	0.64	0.38
African Ancestry	0.09	0.05	0.11	0.05	0.00	0.06
European Ancestry	0.82	0.49	0.60	0.37	0.29	0.52
Head circumference (cm)	55/57	56/57	54/56	55/57	55/57	55/57
Height (cm)	162/175	159/172	160/173	158/172	158/171	160/172
Hip circumference (cm)	98/100	100/102	94/95	95/97	96/99	97/99
Melanin Index	32/32	36/35	34/33	36/35	37/37	34/35
Waist circumference (cm)	75/87	77/90	77/81	81/87	80/88	78/87
Weight (kg)	60/76	61/76	56/70	60/74	56/71	59/74
Male pattern baldness						
(1) No baldness	70	76	88	65	86	72
(2) Some baldness	30	24	12	35	14	28
Graying						
(1) No graying	78/65	80/82	90/87	70/67	92/88	81/78
(2) Some graying	22/35	20/18	10/13	30/33	8/12	19/22
Eye color						
(1) Blue/Grey	8/8	1/3	2/2	1/1	0/0	3/3
(2) Honey	4/2	4/5	10/11	3/2	1/2	5/5
(3) Green	14/15	4/10	8/8	4/6	3/2	7/9
(4) Light brown	19/20	9/9	16/15	21/21	11/8	16/14
(5) Dark brown/Black	55/55	83/74	64/64	72/71	85/87	68/70
Hair color						
(1) Red/reddish	1/1	1/0	1/0	0/0	0/0	1/1
(2) Blond	7/5	3/1	2/2	2/1	1/0	3/2
(3) Dark blond/light brown	35/28	18/11	16/12	21/12	10/5	22/14
(4) Brown/Black	57/65	78/87	81/85	77/86	90/95	75/84
Hair shape						
(1) Straight	41/41	48/62	39/33	46/45	45/41	43/47
(2) Wavy	38/34	38/32	39/38	41/43	42/38	39/37
(3) Curly	18/22	10/4	20/27	12/12	12/20	15/15
(4) Frizzy	3/4	3/1	2/2	1/0	1/1	2/2

Note: Values shown are medians except for categorical traits where the numbers indicate percentages in that category. Data for women is shown in the numerator (except for Male pattern baldness). For the regression analyses (Tables 2 and 3 below) categorical phenotypes 15–17 were considered ordinal variables with 4 or 5 ordered integer levels as specified here (see Methods). Individual ancestry histograms for each country are presented in Text S1.

### Ancestry estimation

We estimated individual African/European/Native American admixture proportions with data for 30 highly informative SNPs using the ADMIXTURE program [17]. These markers were chosen from the 5,000 proposed by Paschou et al (2010) [18] as highly informative for continental ancestry estimation (see Methods). The selected set of markers produced individual ancestry estimates in 372 Colombians, included in a recent genome-wide association study [19], with correlations of ∼70% (for the three continental ancestries) compared to estimates obtained with 50,000 markers (LD-pruned), and identical sample means. Although we estimated individual ancestry with a relatively small number of markers, we verified that the inferences drawn are robust to the level of uncertainty of the estimates obtained (see below).

### Geographic variation of ancestry

**Figure 1:**
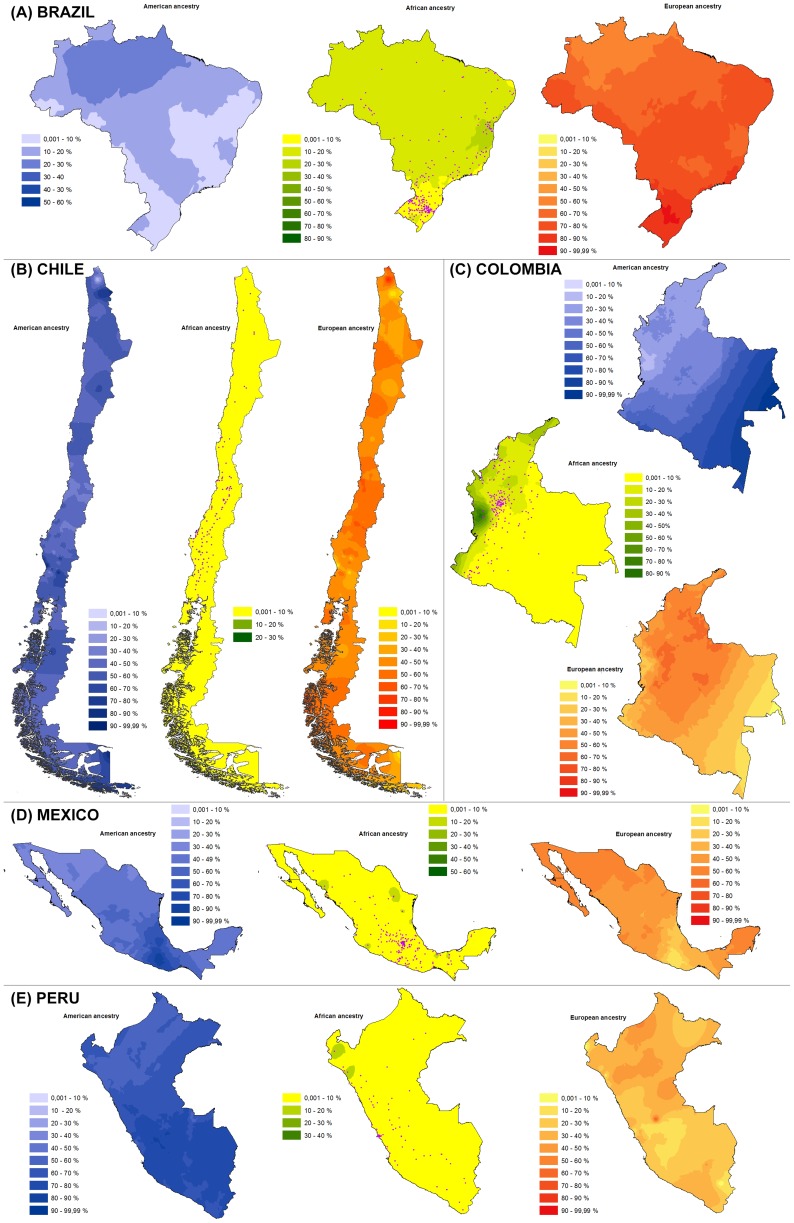
Geographic distribution of Native American (blue), African (green) and European (red) ancestry based on individual estimates for samples from (A) Brazil, (B) Chile, (C) Colombia, (D) México and (E) Perú. To facilitate comparison, color intensity transitions occur at 10% ancestry intervals for all maps. The birthplace of individuals are indicated by purple dots on the African ancestry map. Sampling density is shown in Figure S4. Maps were obtained using Kriging interpolation as detailed in the text.

Consistent with previous studies, we observe extensive variation in ancestry between countries (Table 1) as well as between individuals within countries (Text S1) and between socioeconomic strata (Text S2) [12], [13], [20]–[22]. In order to obtain a spatial representation of variation in ancestry we obtained interpolated maps based on the geographic coordinates for the birthplaces of research volunteers. The geographic distribution of these birthplaces (Figure 1 and Figure S4) overlaps with regional population density from national census data (Figure S5). Consistent with this pattern, the number of volunteers for each birthplace correlates with census size for these localities: Brazil (r = 0.32, p-value <10^−5^), Chile (r = 0.51, p-value <10^−4^), Colombia (r = 0.54, p-value <10^−13^), Mexico (r = 0.44, p-value <10^−8^), Perú (r = 0.41, p-value <10^−4^). Few volunteer birthplaces were thus located in sparsely populated regions (e.g. Amazonia) and geographic interpolation of ancestry in those regions should be regarded with special caution.

The Brazilian sample (Figure 1A) shows widespread European ancestry with the highest levels being observed in the south. African ancestry is also widespread (except for the south) and reaches its highest values in the East of the country. Native American ancestry is highest in the north-west (Amazonia). The Chilean sample (Figure 1B) shows the least regional variation, with low levels of African ancestry throughout the country. European and Native American ancestry are relatively uniform, although somewhat higher European ancestry is seen around the main urban areas of the north and centre, Native ancestry predominating elsewhere, particularly in the south. The Colombian sample (Figure 1C) shows highest African ancestry in the coastal regions (particularly on the Pacific) and highest European ancestry in central areas. Native ancestry appears highest in the south-west and in the east of the country (Amazonia) but interpolations in these areas are based on few data points. In the Mexican sample (Figure 1D) Native American ancestry is highest in the centre/south of the country with the north showing the highest proportion of European Ancestry. African ancestry is generally low across Mexico except for a few coastal regions. The Peruvian sample (Figure 1E) shows substantial Native American ancestry throughout the country, particularly in the south, European ancestry appears highest around northern/central areas. African ancestry in Peru is generally low, except for parts of the northern coast.

To evaluate the statistical significance of the observed spatial variation in ancestry we calculated Moran's Index (I) of association between each individual ancestry component and spatial location. These were significant for the three ancestries in all countries (p-values <0.02). Since the three ancestry components are not independent, we also calculated canonical correlation coefficients between ancestry and geographic location. These were also significant for all countries (p-values <0.001). The variation in ancestry seen in the admixture maps of Figure 1 also result in highly significant correlations of the three ancestries with altitude of birthplace (p-values <2×10^−16^ for the three ancestries): African and European ancestry decreases with altitude (r of −0.24 and −0.39, respectively), while Native American ancestry increases (r = 0.48).

The Kriging interpolation scheme used in building the maps of Figure 1 uses the mean ancestry at each birthplace and does not provide information on the extent of individual variation in ancestry at each map location. In the 102 birthplaces with 10 or more individuals sampled we observe that the standard deviation in the three individual ancestry estimates extends over a wide range: African (0.012–0.022), European (0.046–0.273) and Native American (0.039–0.274). We evaluated the correlation of this variation in individual ancestry with the census size of these localities and found a significant positive correlation for all ancestries (r>0.3, p-values <0.01).

### Phenotypic diversity and genetic ancestry

Regression of phenotypic variation on genetic ancestry (taking Native American as reference) demonstrates a significant effect for most of the traits examined (p-value <10^−3^ using a conservative Bonferroni multiple testing correction, Table 2). Among the non-facial phenotypes (accounting for sex, country, age, educational attainment and wealth) higher European ancestry is associated with: increased height, lighter pigmentation (of hair, skin and eyes) (Figure S6), greater hair curliness and male pattern baldness. Hair graying approaches statistical significance (p-value 10^−2^). Higher African ancestry is associated with: increased height, higher skin pigmentation and greater hair curliness. The proportion of phenotypic variance explained by ancestry is highest for skin pigmentation (19%) followed by hair shape (8%) and color of eyes and hair (4% and 5%, respectively) but at most 1% for the other phenotypes.

**Table 2:** Multiple linear regression of physical appearance traits on European and African ancestry.

	European ancestry		African ancestry			
Trait	Coef.	P-value	Coef.	P-value	%R^2^	% Δ R^2^
1. Weight	0.35	0.70	0.95	0.54	32	0
2. Height	7.31	***2.00E-16***	8.14	***2.00E-16***	55	2
3. Hip circumference	−0.03	0.96	0.18	0.87	11	0
4. Waist circumference	−4.69	***2.50E-09***	−6.46	***2.36E-06***	26	1
5. Head circumference	−0.03	0.88	0.81	0.02	20	3
6. Melanin Index	−10.05	***2.00E-16***	11.89	***2.00E-16***	25	19
7. Balding	0.12	***8.80E-08***	0.15	***8.77E-05***	23	1
8. Hair shape	0.47	***2.00E-16***	2.41	***2.00E-16***	11	8
9. Eye color	−1.26	***2.00E-16***	0.24	0.08	11	5
10. Graying	0.06	0.01	0.11	0.01	44	0
11. Hair color	−0.62	***2.00E-16***	−0.01	0.87	12	5
12. Eye fold	−0.37	***2.00E-16***	−0.37	***1.71E-04***	27	1
13. Centroid Size	−12.98	***7.74E-05***	2.94	0.62	48	0
14. PC-1(19%)	0.02	***1.42E-11***	0.04	***1.24E-12***	15	**2**
15. PC-2(12%)	−0.01	1.00E-03	−0.02	***7.80E-06***	2	0
16. PC-3(10%)	−0.01	***8.85E-13***	0	0.84	21	**3**
17. PC-4(7%)	0.01	***2.34E-08***	−0.02	***6.14E-08***	18	**5**
18. PC-5(7%)	0.01	***3.16E-05***	0	0.6	5	0

Note: All regressions account for age, sex, country, education and wealth. Regressions for facial features (traits 13 to 18) also account for BMI and height. %R^2^ refers to trait variance explained by a regression model incorporating European and African ancestry (being proportions, European, African and American ancestries sum up to 1 and since in this sample African ancestry is very low (median of 7%), we use Native American ancestry as a baseline). %Δ R^2^ refers to the difference in variance explained by this full model and a model without ancestry as a predictor. P-Values <10^−3^ are shown in bold italic. The facial features (traits 13 to 18) refer to morphogeometric summaries of face variation derived from 3D landmark coordinates (see Methods). PC = Principal Components of the procrustes 3D landmark coordinates (% in parenthesis refer to variance explained by that PC).

We also observed highly significant effects of educational attainment (p-value 3.87×10^−13^) and age (p-value <2×10^−16^) on height, with height increasing for individuals born more recently at a rate ∼1 cm every 10 years (Text S3).

Genetic ancestry also has a range of effects on facial features, both in terms of size and shape, after accounting for height and BMI (in addition to the other covariates). Higher European ancestry is associated with reduced eye fold and an overall smaller face (centroid size).

Face size and shape effects were also evaluated through the analysis of all pair-wise inter-landmark distances (Table S3). Amongst these distances, 133 and 2 show significant effects of European and African ancestry, respectively (p-values 10^−6^ assuming a conservative Bonferroni multiple testing correction; Table S3). The most significant effects of European ancestry (P<10^−10^) involve mainly distances between landmarks placed on the lips and nose. Face shape variation, independent of size, was assessed via Principal Components (PCs) of procrustes 3D coordinates. Significant effects of European ancestry were detected for PCs 1 and 3–5, while African ancestry impacts on PCs 1, 2 and 4 (Table 2, Text S5 and Figure S3). These 5 PCs account for ∼55% of the variation in face shape captured by the 36 landmarks placed on the facial photographs, with ancestry explaining up to 5% of the variance in PC scores (for PC4). Examination of the correlation between inter-landmark distances and facial PCs, indicates that the highest correlation of distances between landmarks of the lips and nose is with PC4 (results not shown), consistent with this PC showing the largest proportion of variance explained by ancestry (Table 2).

### Genetic ancestry, phenotypic diversity and self-perception

**Figure 2:**
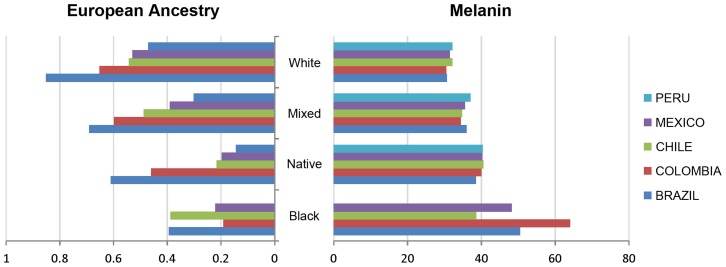
Bar plots contrasting skin pigmentation (Melanin Index) to proportion of European genetic ancestry across four self-identified ethno/racial categories in samples from Brazil, Chile, Colombia, México and Perú. Sample sizes and all estimates of pigmentation and ancestry, are presented in Table S4. In Perú no individual self-identified as “Black”.

Four ethno/racial categories (“Black”, “White”, “Native” and “Mixed”) are commonly used across Latin America in national censuses and other population surveys. We contrasted genetic ancestry and skin pigmentation (as measured by the melanin index) across these four self-estimated categories for the countries sampled (Figure 2 and Table S4). Within each country there is a gradient of decreasing European ancestry (and increasing pigmentation) for the “White”, “Mixed” and “Native/Black” categories. Across countries, skin pigmentation is relatively uniform within ethnicity categories, except for “Black”. For “White”, “Native” and “Mixed” the mean melanin index across countries varies within ∼2 units, while the range for “Black” is ∼25 units. By contrast, genetic ancestry varies greatly between countries for all ethnicity categories. For example, European ancestry varies across countries by about 40% for “White”, “Mixed” and “Native” and about 20% for “Black” (Figure 2; estimates for African and Native American ancestry are shown in Table S4).

**Figure 3:**
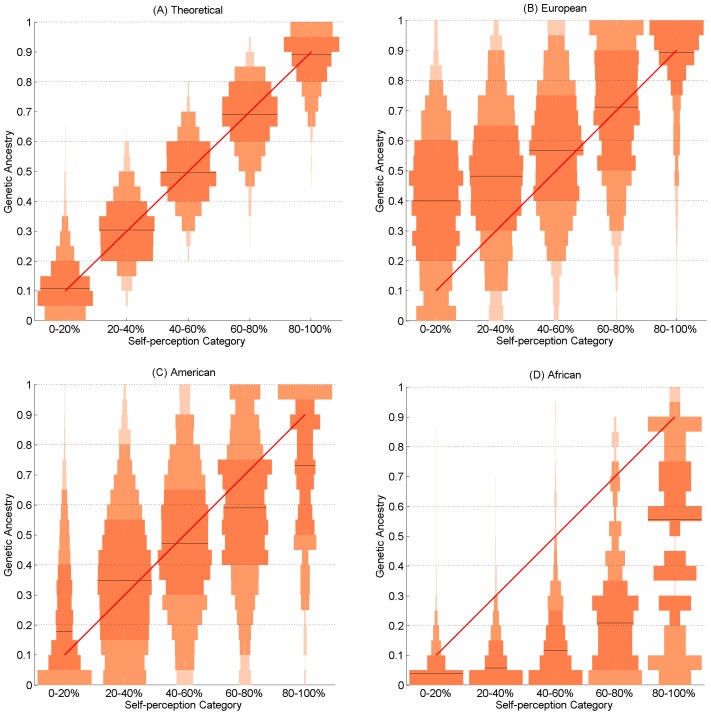
Vertical histograms (pyramid plots) showing the distribution of genetic ancestry for each of the five self-perceived ancestry categories. As reference, (A) shows the theoretical case of agreement between self-perceived and genetically estimated ancestry. For this plot random values were drawn from a beta distribution such that, for each self-perception band, the median ancestry lies at the centre of a 0.2 interval containing 75% of the simulated ancestry values. The number of simulated values was fixed at our sample size. Panels (B), (C) and (D) show respectively, the observed distributions for European, Native American and African ancestries. The red diagonal line indicates the midpoint, on the genetic ancestry scale, of each self-perceived ancestry category. Distributions are coded in three shades of orange: the darkest shade denotes the central quartiles (the median shown as a brown line), the medium-shade indicates the 5%–95% range, and the lightest shade refers to samples outside this range. For European ancestry, self-perception tends to underestimate genetic ancestry (the distributions are mostly above the diagonal). By contrast, self-perception tends to overestimate African ancestry (the distributions are mostly below the diagonal). At increasing levels of Native American genetic ancestry self-perception first underestimates then overestimates genetic ancestry (the distributions are on both sides of the diagonal). Simulations and plots were carried out using MATLAB [61].

Contrasting self-perceived (ranked into five bands at 20% increments) and genetically estimated continental ancestry we observe a moderate, but highly significant, correlation: America: r = 0.48, P<2.2×10^−6^, Europe: r = 0.48, P<2.2×10^−6^, Africa: r = 0.32, P<2.2×10^−6^. However, there is a trend for higher self-perceived Native American and African ancestry to exceed the genetic estimates (Figure 3). Similarly, there is a trend for lower self-perceived Native American and European ancestry to underestimate the genetic ancestry (Figure 3). To explore these trends further we performed a multiple linear regression of the difference between self-perceived and genetically estimated ancestry (i.e. the bias, see Methods), using genetic ancestry and covariates as predictors (Table 3). As expected, we observe that genetic ancestry has a highly significant effect (<2×10^−16^ for all ancestries) and the negative sign of the regression coefficients reflects the orientation of bias seen in Figure 3. At increasing European genetic ancestry, there is greater underestimation in self-perception (a more negative bias). By contrast, with increasing African genetic ancestry there is less overestimation (less positive bias). For Native American ancestry, there is an overestimation (positive bias) at low levels, and an underestimation at high levels of ancestry (negative bias).

**Table 3:** Multiple linear regression of the difference (Δ) between self-perceived and genetically estimated ancestry for the three continental components.

	Δ AFRICA		Δ AMERICA		Δ EUROPE	
	Coef.	P-value	Coef.	P-value	Coef.	P-value
European Ancestry*	−0.01	0.48	−0.45*	<2.00E-16*	−0.52	***<2.00E-16***
African Ancestry	−0.23	***<2.00E-16***	−0.07	0.02	0.07	0.04
Country-Chile	−0.05	***1.48E-12***	1.59E-03	0.85	−0.03	1.51E-03
Country-Colombia	−0.03	***7.29E-08***	0.04	***9.80E-08***	−0.09	***2.00E-16***
Country-México	−0.05	***7.50E-09***	0.02	0.01	2.95E-03	0.79
Country-Perú	−0.35	***2.43E-05***	−0.03	0.78	−0.80	***2.27E-11***
Wealth	−1.45E-04	0.81	2.63E-04	0.72	2.91E-03	***6.41E-04***
Education	−2.17E-03	0.45	3.96E-03	0.25	1.56E-03	0.69
Age	1.75E-04	0.59	8.45E-04	0.03	7.87E-04	0.08
Sex	−0.01	0.03	1.40E-03	0.83	0.02	1.16E-03
Balding	0.01	0.14	0.01	0.29	−0.01	0.39
Hair graying	−0.01	0.32	−0.02	4.23E-03	−0.01	0.27
Height	−3.79E-04	0.19	−7.35E-04	0.04	1.81E-03	***6.19E-06***
Melanin index	2.55E-03	***8.97E-12***	2.97E-03	***7.91E-11***	−0.01	***2.00E-16***
Hair shape	0.02	***2.06E-13***	−0.01	***4.81E-04***	−2.93E-03	0.38
Eye color	4.26E-03	0.02	0.01	***2.04E-07***	−0.02	***1.40E-11***
Hair color	0.01	0.01	0.02	***7.11E-04***	−0.03	***3.59E-08***
Eyefold	0.00	0.68	0.01	4.01E-03	−0.02	***3.98E-04***
PC1	0.10	0.36	−0.30	0.03	0.33	0.03
PC2	−0.10	0.44	−0.03	0.87	0.03	0.87
PC3	0.38	0.02	0.31	0.12	−0.98	***6.39E-06***
PC4	−0.45	0.02	−0.03	0.91	0.91	***5.03E-04***
PC5	−0.03	0.86	0.35	0.10	0.14	0.57

NOTE: Δ refers to self-perception (bands 1 to 5, see Methods) minus continental ancestry proportions (0–100%) estimated from the genetic data. Sex and country of sampling were incorporated in the analyses as factors while the other variables were treated as quantitative. For ease of interpretation, the regression coefficient and p-value for Δ AMERICA (*) refer to Native American (not European) ancestry.

Most of the phenotypic traits that show ancestry effects (Table 2) also have a significant effect on self-perception bias (Table 3). There is a particularly strong effect of pigmentation: individuals with lower skin pigmentation tend to overestimate their European ancestry while individuals with higher pigmentation overestimate their Native American and African ancestries. Similarly, lighter eye and hair color lead to an overestimation of European ancestry and an underestimation of Native American ancestry (but not African ancestry). Hair type is strongly associated with an overestimation of African ancestry. Marginally significant associations are seen with other phenotypes, including facial features such as eye fold (leading to an underestimation of European ancestry) and landmark coordinate PCs (Table 3). An effect of social factors on perception bias is evidenced by the observation that greater wealth is significantly associated with an overestimation of European ancestry and that there is significant variation in bias between countries (Table 3). We examined the impact on these results of the uncertainty associated with the ancestry estimates by repeating the regression analyses using ancestry estimates obtained with a subset of 15 markers (Methods). We found that the same covariates had significant effects and that the regression coefficients were not significantly different in the two sets of regression analyses.

## Discussion

Since the late 15^th^ century, the population of what is now called “Latin America” has undergone major demographic changes within the context of a highly diversified physical and social environment [6], [23]. These changes include the occurrence of waves of immigration from various parts of Africa and Europe, the resulting decline of the Native populations most exposed to the immigrants and a variable admixture between these groups. There have also been a number of noticeable population movements in the region. For example, in recent generations there has been an extensive migration to the cities, Latin America now being the most urbanized region of the world (about 80% of its population is currently considered urban) [24]. Three of the countries we sampled (Brazil, Mexico and Colombia) are the most populous in the region and the combined population of the five countries examined here account for ∼70% of Latin Americans. Although ours is a convenience sample, the individuals studied show considerable variation in birthplace and for a range of biological and social variables, illustrating the extensive heterogeneity of Latin Americans.

The interpolated ancestry maps obtained (Figure 1) are consistent with other genetic studies [20], [21], [25], [26] and with census information on the distribution of the main ethnicity groups within each country (available at www.ine.cl; geoftp.ibge.gov.br; www.igac.gov.co; www.censo2010.org.mx, www.indepa.gob.pe). Altogether, these data underline the extensive genetic structure existing within and between Latin American countries. It is possible to relate this genetic heterogeneity to well documented historical factors [6], [23], [27]. Broadly, Native American ancestry is highest in areas that were densely populated in pre-Columbian times (particularly Meso-America and the Andean highlands) as well as in regions that received relatively little non-native immigration and which currently have relatively low population densities (e.g. Amazonia). During the colonial period Africans were brought to Latin America as forced labour mainly to coastal tropical areas, particularly in the Caribbean and Brazil [28]. That country was the main recipient of African slaves in the region (representing about 40% of all African slaves brought to the Americas [29]). Early (mostly male) Iberian immigrants settled across the continent, admixing extensively with Native Americans and Africans [5]. These were followed by further currents of European immigration, including individuals from various parts of Europe (often arriving as a result of governmental initiatives) and resulting in the dense settlement of specific geographic regions (such as the south of Brazil). The larger variance in individual ancestry observed for larger urban centres is consistent with the increasing urbanization of Latin America seen recent generations, the cities absorbing immigrants with diverse genetic backgrounds. Other than demographic history, it is possible that assortative mating has also contributed to shaping population structure across Latin America. The Iberian “Conquest” (i.e. the first century of settlement) was characterized by extensive admixture between Natives and immigrants (driven by the highly predominant immigration of males) [5]. However, during the subsequent colonial period society became increasingly stratified, including the instauration during the 18^th^ century of a caste system regulating marriages [6], [27]. These restrictions were mostly abolished with the establishment of republican governments in the 19^th^ century [6]. However, a number of studies have documented continuing assortative mating in Latin America, in relation to genetic ancestry, physical appearance and a range of social factors [30]–[34].

The pattern of variation we observe between physical appearance and genetic ancestry is consistent with information on the variation in frequency of the traits examined in Native Americans, Europeans and Africans. Constitutive skin pigmentation (i.e. in areas not exposed to light), hair and eye color and hair type are traits with little environmental sensitivity and show large differences between continental populations [35]. As expected, increased European ancestry shows a highly significant association with lighter skin, hair and eye pigmentation. A number of allelic variants impacting on these traits have been identified in Europeans and certain of these show large allele frequency differences between Europeans and non-Europeans [1], [2], [36]. We also found a highly significant effect of ancestry on hair type, individuals with higher Native American ancestry showing greater frequency of straight hair, a phenotype that is essentially fixed in Native Americans. Recent studies in East Asians implicate a p.Val370Ala substitution in the EDAR gene in hair morphology [37]–[39]. One of the ancestry informative markers typed here (rs260690) is located in the first intron of *EDAR*, is in high linkage disequilibrium with the p.Val370Ala variant in the HapMap dataset and is strongly associated with hair type in our sample, after accounting for ancestry (Text S4), suggesting that variants at *EDAR* could be impacting on hair morphology in Latin Americans. Greater European ancestry also correlates significantly with higher rates of male balding and (marginally) with hair greying (our sample is perhaps underpowered to detect these effects due to its relatively young age; Table 1). Although no thorough comparative data is available, classical population studies indicate that hair greying and androgenetic alopecia are rarer, less severe and of later onset in Native Americans than in other continental populations [40] and our data points to the existence of loci influencing the continental distribution of these traits. Studies in Europeans have recently identified loci associated with androgenetic alopecia [41], [42], but no similar analyses have been performed for hair greying.

Recent genome-wide association analyses in Europeans have implicated loci for variation in height and related anthropometric traits [43], [44]. However, these traits are also strongly influenced by environmental factors, including nutrition [45]. In the sample studied here we find that Native American ancestry correlates significantly with lower height and we also detect a significant effect of socioeconomic position (Text S3), lower socioeconomic position correlating with decreased height. The significant effect of age on height, with younger individuals tending to be taller than older ones suggests that the two socioeconomic indicators examined here (education and wealth) capture only part of the environmental variation impacting on height. The rate of increase in height for individuals born more recently (∼0.1 cm/year) estimated here is similar to that obtained from extensive longitudinal surveys in Latin America (∼1 cm per decade in the last century), an observation that has been interpreted as resulting from the historical improvement in living standards across the region [45], [46]. It is thus possible that the ancestry effect on height that we detect could be influenced by environmental factors that correlate with ancestry that are not captured by the socioeconomic variables examined here.

The ancestry effects that we detect for facial features (eye fold, face shape and size), but not for head circumference, agree with the notion of a greater developmental and evolutionary constraint on neuro-cranium than on facial variation. This is also in line with proposals that human facial features include a range of environmental adaptations [47]–[49]. Aspects of face shape variation captured by principal components analysis that are influenced by genetic ancestry include mainly, width and height of the face, facial flatness, position of the glabella and fronto-temporal points, extent of eye fold and the relative size and position of lips and nose (a fuller description of face shape variation associated with each PC is presented in Text S5 and Figure S3). Two genome-wide association scans in Europeans have identified a few loci associated with aspects of face shape [50], [51] but these results are pending confirmation by further studies. No genetic variants have yet been implicated in intercontinental differentiation for facial features.

Our joint analysis of genetic, phenotypic and self-perception variation emphasizes the strong impact of physical appearance on self-perception. Comparison of skin pigmentation across self-perceived ethno/racial categories shows remarkable consistency between countries, underlining the weight given to this trait in self-perception [52]. The large variation in genetic ancestry between countries for each ethnicity category illustrates the relatively low predictive power of physical appearance for genetic ancestry. Although we detected highly significant effects of ancestry on many of the phenotypes examined, the observed correlations are relatively low (Table 2). The poor reliability of physical appearance as an indicator of genetic ancestry likely relates to the impact of environmental variation on some of these traits, and to their specific genetic architecture. Particularly, a few genetic variants could have relatively large phenotypic effects (as documented for pigmentation [2], [36]). The impact of physical appearance on self-perception of ancestry likely relates to admixture in Latin America largely occurring many generations ago and the frequent unavailability of reliable genealogical information. The contrast between self-perceived and genetically estimated admixture proportions confirms the impact of physical appearance on self-perception and shows how certain traits, particularly but not exclusively related to pigmentation, can bias self-perception of ancestry. This biased perception of physical attributes is likely to be influenced by social and individual factors shaping the interpretation of phenotypic variation. The effect of such factors is illustrated by the observation of differences in bias across countries and the positive correlation between wealth and European ancestry (Table 2). An effect of wealth on self-perception of ancestry has also been the subject of study in the sociological literature on Latin America [52].

In conclusion, our study sample illustrates the extensive geographic variation in genetic ancestry seen across Latin America, reflecting the heterogeneous demographic history of the region. The highly significant impact of genetic ancestry on physical appearance is consistent with some of the phenotypic variation seen in Latin Americans stemming from genetic loci with differentiated allele frequencies between Africans, Europeans and Native Americans [53]. Further analysis of the study sample collected here should enable the identification of such loci. The significant correlation between self-perceived and genetically estimated ancestry is consistent with the observed effects of genetic ancestry on physical appearance. However, self-perception is biased, possibly due to non-biological factors affecting the perception of phenotypic variation and to the genetic architecture of physical appearance traits. Our findings exemplify the informativity of Latin America for studies encompassing genetic, phenotypic and sociodemographic information and the interest of a multidisciplinary approach to human diversity studies.

## Materials and Methods

### Study subjects

Recruitment took place mainly in five locations: México City (México), Medellín (Colombia), Lima (Perú), Arica (Chile) and Porto Alegre (Brazil). With the exception of Chile, most subjects recruited in these cities were students and staff from the universities participating in this research. In Chile about 2/3 of the subjects recruited were professional soldiers. In Brazil ∼10% of samples were collected in smaller towns of the states of Rio Grande do Sul, Bahia and Rondonia. Adult subjects of both sexes were invited to participate mainly through public lectures and media presentations. Maps showing the number of volunteers in each unique birthplace are presented in Figure S4. Being a convenience sample, the main collection sites are overrepresented on these maps for each country. We obtained ethics approval from: Escuela Nacional de Antropología e Historia (México), Universidad de Antioquia (Colombia), Universidad Perúana Cayetano Heredia (Perú), Universidad de Tarapacá (Chile), Universidad Federal do Rio Grande do Sul (Brazil) and University College London (UK). All participants provided written informed consent. Blood samples were collected by a certified phlebotomist and DNA extracted following standard laboratory procedures.

### Phenotypic data

A physical examination of each volunteer was carried out by the local research team using the same protocol and instruments at all recruitment sites. We obtained: height, weight, head, hip and waist circumference, cheilion-cheilion width and sellion-gnation height. All measurements were obtained in duplicate and the mean of the two measurements retained for further analyses. We recorded eye colour into five categories (1-blue/grey, 2-honey, 3-green, 4-light brown, 5-dark brown/black), and natural hair colour into four categories (1-red/reddish, 2-blond, 3-dark blond/light brown or 4-brown/black). Balding in males was recorded using a modified Hamilton scale as: 0) no hair loss, 1) frontal baldness only, 2) frontal hair loss with mild vertex baldness, 3) frontal hair loss with moderate vertex baldness, and 4) frontal hair loss with severe vertex baldness. Similarly, graying was recorded along a five point scale: 0) for no greying, 1) for predominant non-graying, 2) for ∼50% graying, 3) for predominant greying and 4) for totally white hair. Due to the small number of individuals in categories 1–4 for male pattern balding and greying, we pooled these categories so as to contrast only two categories (presence or absence of the trait). Macroscopic hair type was categorized by visual inspection as 1-straight, 2-wavy, 3-curly or 4-frizzy. A quantitative measure of constitutive skin pigmentation (the Melanin Index) was obtained using the DermaSpectrometer DSMEII reflectometer (Cortex Technology, Hadsund, Denmark). Measurements were obtained from both inner arms and the mean of the two readings used in the analyses.

Five digital photographs of the face: left side (−90°), left angle (−45°), frontal (0°), right angle (45°), right side (90°) were taken from ∼1.5 meters at eye level using a Nikon D90 camera fitted with a Nikkor 50 mm fixed focal length lens. The frontal facial photographs were used to score (by visual inspection) the presence of an eye fold along the upper eye lids using a three point scale: 0) absence 1) partial (interior, middle or outer fold) and 2) full (along the entire eye lid).All photographs were annotated manually with 36 anatomical landmarks and 3D landmark coordinates extracted using the software Photomodeler (http://www.photomodeler.com/ Eos Systems Inc, Vancouver, Canada) (Figure S1). Landmark configurations were superimposed by Generalized Procrustes Analysis [54] and Principal Components (PCs) of the 3D landmark coordinates extracted using the software MORPHOJ [54]. To ease visualization of the 3D shape changes associated with each PC we obtained deformation surfaces via a thin plate spline algorithm.

### Socioeconomic information and self-perception of ancestry

A structured questionnaire was applied to each volunteer. We obtained information on two indicators of socioeconomic position (Text S2). The first indicator is highest education level attained, categorized as: (1) none/primary/technical, (2) secondary and (3) university and post-graduate. The second indicator is a wealth index obtained from a list of items used to assess living standards. These items were: home ownership, number of bathrooms at the place of residence, ownership of household items (cars, bicycles, fridge/freezer/dishwasher, TVs, radios, CD/DVD players, vacuum cleaner, washing machine) and availability of domestic service. We used polychoric principal component analysis to examine the variability of each country sample and retained the first principal component as an indicator of wealth. To allow comparisons across countries we converted an individual's wealth score to decile within each country.

The questionnaire included items exploring self-perception of ethnicity in the categories: “Black”, “Native”, “White” and “Mixed”, and self-perception of African, European, and Native American ancestry proportions. This was explained as a personal estimation of the proportion of ancestors that had a particular continental origin. We proposed a five point scale, expressed in 20% per cent brackets (and in words): 1) 0–20% (none or very low), 2) 20–40% (low), 3) 40–60% (moderate), 4) 60–80% (high) and 5) 80–100% (very high or total). The questionnaire also recorded information on the place of birth of the volunteer.

### Genetic admixture estimation

In order to select 30 markers highly informative for estimating African/European/Native American ancestry, we started from the list of 5,000 markers, highly informative for world-wide continental ancestry estimation, identified by Paschou et al (2010) [18] using the approach of Rosenberg et al. (2003) [55] based on the worldwide CEPH-HGDP cell panel genotyped with Illumina's Human610-Quad beadchip (including data for about 600,000 SNPs [56]). The full list of these 5,000 markers is at: http://www.cs.rpi.edu/~drinep/HGDPAIMS/WORLD_5000_INFAIMs.txt. Of these, allele genotype data is available in Native Americans for 3,848 markers [57], of which 2,392 have been placed on subsequent Illumina bead-chip products. This subset of markers was retained for selection of those to be typed here so as to facilitate subsequent data comparison and integration. We ranked these 2,392 markers based on allele frequency differences in European-Native American or European-African samples. Amongst markers with the highest inter-continental allele-frequency differences we selected those with lowest heterozygosity in Native Americans (so as to reduce the effect of variable allele frequencies between Native Americans on ancestry estimation). Of the final set of 30 markers retained, 13 are monomorphic in 408 Native Americans (from 47 populations from México Southwards [57]), the rest have minor allele frequencies ranging from 0.01 to 0.15 (median 0.06) in that group of populations. The list of markers typed is provided in Table S1. Genotyping was carried out by LGC genomics (www.lgcgenomics.com/). In a sample of Colombians recently included in a genome-wide association study that used Illumina's 610 chip [19], this set of 30 markers produced individual ancestry estimates with correlations of ∼0.7 (for all the three ancestries) compared with ancestry estimates obtained using an LD-pruned set of 50,000 markers from the chip data, and identical mean estimates. We compared the accuracy of these estimates with estimates obtained using markers from the list of 446 proposed by Galanter et al. (2012) [58], specifically for studying admixture in Latin Americans. From this list, 152 markers are present on Illumina's 610 chip (i.e. ∼5 times the number of markers that we used) and produced estimates with correlations of ∼0.85 with the ancestry estimates from the 50,000 marker set. By contrast, when the set of markers we selected was reduced to 15, the resulting ancestry estimates had a correlation of ∼0.6 with the 50,000 marker set estimates, again showing that there is a diminishing return in accuracy when one increases the numbers of SNPs used in ancestry estimation.

Individual African, European and Native American ancestry proportions were estimated using the ADMIXTURE program [17] using supervised runs where African, European and Native American reference groups (K = 3) were provided (see below). Unsupervised runs at K = 3 produced very similar estimates (Figure S2), confirming our choice of ancestry-informative markers and parental populations. Standard errors of the individual ancestry estimates were obtained by bootstrap using the program's default parameters (200 replication runs). Data from a total of 876 individuals sampled in putative parental populations were used in ancestry estimation and specified in the supervised ADMIXTURE runs. These were selected from HAPMAP, the CEPH-HDGP cell panel [56] and from published Native American data [57] as follows: 169 Africans (from 5 populations from Sub-Saharan West Africa), 299 Europeans (from 7 West and South European populations) and 408 Native Americans (from 47 populations from México Southwards). The full list of the putative parental population samples (and their sizes) is provided in Table S2.

### Geographic analyses

The birthplace names of all individuals was consolidated into a list of unique locations organized into three fields: city/municipality, region/state and country. Geographic coordinates (and altitude) for each placename were obtained via the Google Maps Geocoding API (https://developers.google.com/maps/documentation/geocoding/). The GeodesiX software (http://www.calvert.ch/geodesix/) was used for the geocoding query. We used the Global Rural-Urban Mapping Project version 1 data set (GRUMPv1; http://sedac.ciesin.columbia.edu/data/set/grump-v1-settlement-points) to attribute census size to these localities (see Supp. Text S6) [59]. We use census data for 1990, as the median age in our country samples ranges between 20 and 25.

Geographic maps displaying spatial variation in individual admixture were obtained with Kriging interpolation using the software ArcGis 9.3 (http://www.esri.com/software/arcgis). The cartographic database was geo-referenced to the SIRGAS geodesic system (Geocentric Reference System for the Americas, www.ibge.gov.br/home/geociencias/geodesia/sirgasing/index.html) using a Universal Transverse Mercator projection. Corel-DRAW X3 (Corel Corporation, Ottawa, Canada) was used to edit the map images. When a geographic location had multiple data entries (i.e. volunteers), the Kriging interpolation scheme uses the mean ancestry at that location. The correlation between the standard deviation of individual ancestry variation (at locations with more than 10 samples) and census size was tested using Spearman's rank correlation (as population sizes are generally non-normal but rather distributed exponentially). Statistical significance was obtained via permutation of individual birthplaces.

We tested the null hypothesis of ancestry being spatially uniformly distributed using two approaches. Firstly, we obtained Moran's ‘I’ index for each ancestry component (African, European, Native American) separately. This index tests for spatial uniformity of a variable using standard autocorrelation models and we evaluated significance by permuting birthplace locations for every individual maintaining constant the number of individuals sampled per location. To assign a single value to each location we used the average ancestry, recalculating this average after every permutation. Secondly, we used canonical correlation analysis. A disadvantage of Moran's method is that the three ancestry variables are not independent, complicating the interpretation of p-values. Canonical correlation allows one to combine the three ancestries into a single variable: it is the maximal correlation between two sets of linear combinations of multiple variables. In our case, the three ancestries constitute one set and the geographical coordinates (latitude & longitude) constitute the second set. Adding quadratic and cubic powers of the geographic coordinates improved the fit, consistent with the curved shape of the ancestry gradients and the existence of regions with markedly different ancestry. Adding a fourth power did not improve the fit any further. P-values were obtained by permutation as above.

### Statistical analyses

To evaluate the effect of ancestry on phenotype we used multivariate regression models including basic covariates (age, sex, country, education, wealth, and optionally BMI and height). Depending on the trait we used multiple linear (for continuous and ordinal traits) or logistic (for binary traits) regression. The categorical traits in Table 2 were considered ordinal variables (converted into four or five integer levels as specified in Table 1). The justification for doing so is the convention that for an ordinal variable with several categories there is little difference in fitting a linear regression model or an ordered probit model [48]. This is true because for these traits we can assume an underlying continuous variable (for eye or hair colour it could be the amount of pigment, for hair shape it could be the curvature of hair). Since an underlying continuous variable converted into ordered categories is the main assumption for the development of a probit model, this similarity in the two analysis holds. We verified this by examining both models and verifying that the results are similar.

Regression results corresponding to the ancestry variables are presented in Table 2 along with R^2^ from this full model. A baseline regression model with only the covariates was also performed, leaving out ancestry, and the difference in R^2^ in the two models was taken to be the proportion of variance in the phenotype explained by ancestry. Standard errors of the individual ancestry estimates (provided by the ADMIXTURE software) were incorporated in the multivariate regressions via the errors-in-variables model [49]. This adjusts the estimated regression coefficients and p-values for all covariates. The error in estimating a variable generally leads to an underestimation of the regression coefficients. However, the p-value still approaches zero under the alternative hypothesis, provided samples sizes are sufficiently large. For the ancestry estimates, the error in estimation was relatively low (∼1–5%), consequently for our large sample sizes the reduction in effect size for each variable was modest (∼5–10%).

To evaluate the relationship between self-perceived and genetically estimated ancestry we performed a bias analysis. This bias was defined as self-perception minus the estimated genetic ancestry. Overestimation therefore means that self-perception exceeds the genetic estimate, while underestimation indicates that self-perception is lower than the genetic estimate. Each genetic ancestry estimate was obtained as a percentage (proportion), while self-perception was recorded into five bands at intervals of 20%. The bias in self-perception was therefore considered zero if the percentage of genetic ancestry fell within the chosen self-perception interval. Otherwise, bias was measured to be the distance of the closest boundary of the self-perception interval to the genetic ancestry percentage. We then performed multivariate linear regression of the bias on the genetic ancestry estimates and other variables (Table 3). The advantage of analysing the bias is that the regression model is easily interpretable. If self-perception was accurate (bias of zero) all the regression coefficients would be non-significant. If the bias is non-zero and some variables show significant effects, the signs of the coefficients are interpretable as leading to overestimation (positive coefficients) or underestimation (negative coefficients) of ancestry, as indicated above.

All statistical analyses were performed using R (www.r-project.org) [60] or MATLAB [61].

## Supporting Information

Figure S1 Position of facial landmarks.
(DOCX)

Figure S2 (A) Supervised and (B) Unsupervised ADMIXTURE runs with K = 3.
(DOCX)

Figure S3 PCs 1–5 obtained from 3D coordinates of facial landmarks, represented by (A) Scatterplots and (B) Facial morphs.
(DOCX)

Figure S4 Birthplace maps of study volunteers in (A) Brazil, (B) Chile, (C) Colombia, (D) México and (E) Perú.
(DOCX)

Figure S5 Populated locations (points) and Population sizes in (A) Brazil, (B) Chile, (C) Colombia, (D) México and (E) Perú.
(DOCX)

Figure S6 Scatterplots of (A) Skin pigmentation (Melanin Index) and (B) Height (in cm), and European genetic ancestry.
(DOCX)

Table S1 Allele frequencies at 30 SNP markers selected for African, European and Native American ancestry estimation and the absolute difference in reference allele frequency (Δ) between continental populations.
(DOCX)

Table S2 Population samples used for genetic ancestry estimation.
(DOCX)

Table S3 Coefficients and P-values for regression of pair-wise inter-landmark distances and ancestry.
(DOCX)

Table S4 Frequency (and %) of four ethnicity categories in each country, mean individual genetic ancestry and Melanin Index in each category.
(DOCX)

Text S1 Variation in ancestry across countries and individuals.
(DOCX)

Text S2 Correlation of socioeconomic position with ancestry.
(DOCX)

Text S3 Regression of height (in cm) on ancestry and covariates.
(DOCX)

Text S4 Regression of hair shape on marker rs260690 and covariates.
(DOCX)

Text S5 Description of face shape changes associated with PCs 1–5 of 3D landmark coordinates.
(DOCX)

Text S6 Obtaining population size at individual birthplaces.
(DOCX)
