# Supplementary material for: Admixture in Latin America: Geographic Structure, Phenotypic Diversity and Self-Perception of Ancestry Based on 7,342 Individuals
Source: PLoS Genet. 2014 Sep 25;10(9):e1004572. doi: 10.1371/journal.pgen.1004572 (PMC4177621; doi:10.1371/journal.pgen.1004572)
Supplement: Figure S1 — Position of facial landmarks. (DOCX) [file pgen.1004572.s001.docx]

## Supplementary Figure S1: Position of facial landmarks.


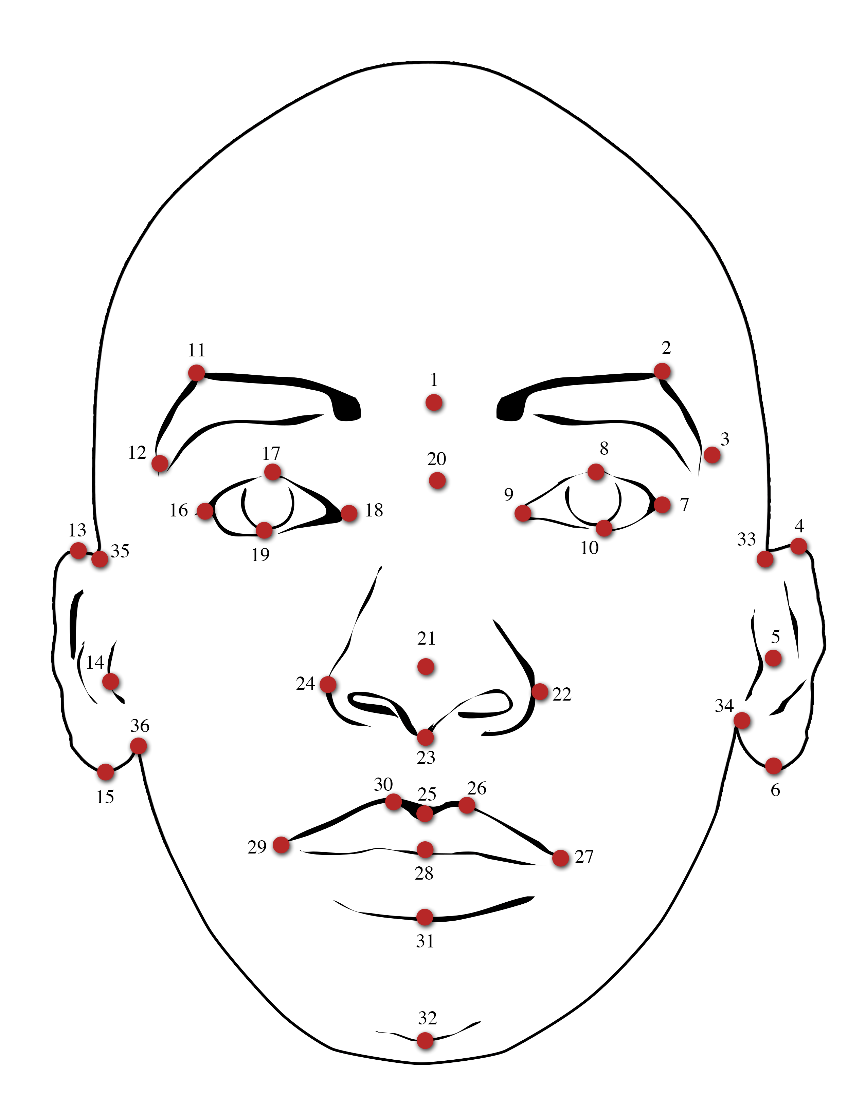


| **Landmarks:**   1. Glabella 2. Superciliare (left) 3. Frontotemporale (left) 4. Superaurale (left) 5. Superior lateral tragion (left) 6. Subaurale (left) 7. Exocanthion (left) 8. Palpebrale superiorus (left) 9. Endocanthion (left) 10. Palpebrale inferiorus (left) 11. Superciliare (right) 12. Frontotemporale (right) 13. Superaurale (right) 14. Superior lateral tragion (right) 15. Subaurale (right) 16. Exocanthion (right) | 1. Palpebrale superiorus (right) 2. Endocanthion (right) 3. Palpebrale inferiorus (right) 4. Sellion 5. Infrapronasale 6. Alare (left) 7. Subnasale 8. Alare (right) 9. Labiale superiorus (sagital) 10. Labiale superiorus (left) 11. Cheilion (left) 12. Stomion 13. Cheilion (right) 14. Labiale superiorus (right) 15. Labiale inferiorus 16. Pogonion 17. Otobasion superiorious (left) 18. Otobasion inferiorous (left) 19. Otobasion superiorious (right) 20. Otobasion inferiorous (right) |
| --- | --- |
