# Supplementary material for: Admixture in Latin America: Geographic Structure, Phenotypic Diversity and Self-Perception of Ancestry Based on 7,342 Individuals
Source: PLoS Genet. 2014 Sep 25;10(9):e1004572. doi: 10.1371/journal.pgen.1004572 (PMC4177621; doi:10.1371/journal.pgen.1004572)

## Supplementary Figure S2: (A) Supervised and (B) Unsupervised ADMIXTURE runs with K=3.

(A) Supervised run where European (Eu: orange), Native American (Am: blue) and African (Af: green) samples define the three populations.


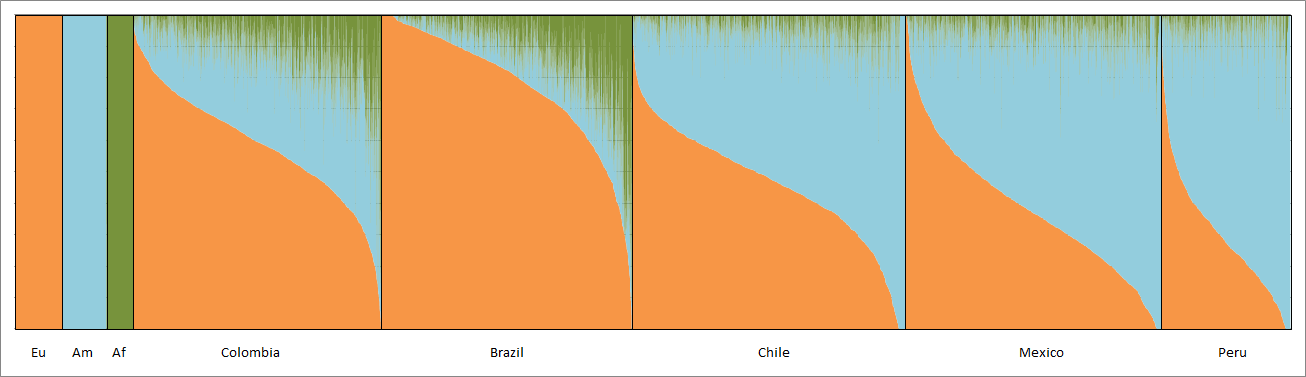


(B) Unsupervised run with the major component in each continental population group colored as above.


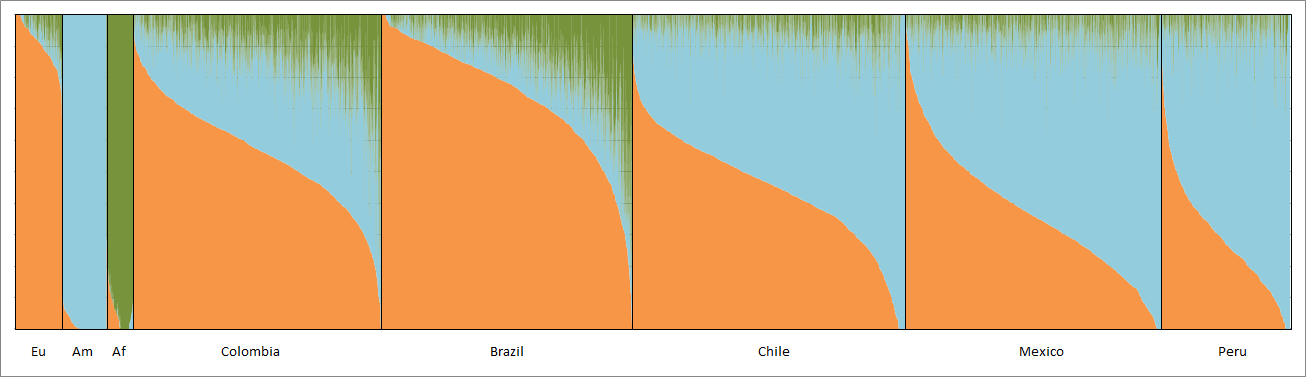

Supplement: Figure S2 — (A) Supervised and (B) Unsupervised ADMIXTURE runs with K = 3. (DOCX) [file pgen.1004572.s002.docx]
