# Supplementary material for: Admixture in Latin America: Geographic Structure, Phenotypic Diversity and Self-Perception of Ancestry Based on 7,342 Individuals
Source: PLoS Genet. 2014 Sep 25;10(9):e1004572. doi: 10.1371/journal.pgen.1004572 (PMC4177621; doi:10.1371/journal.pgen.1004572)

## Supplementary Figure S3: PCs 1-5 obtained from 3D coordinates of facial landmarks, represented by (A) Scatterplots and (B) Facial morphs.

These PCs were obtained from Procrustes landmark coordinates after accounting for BMI and sex.

1. **
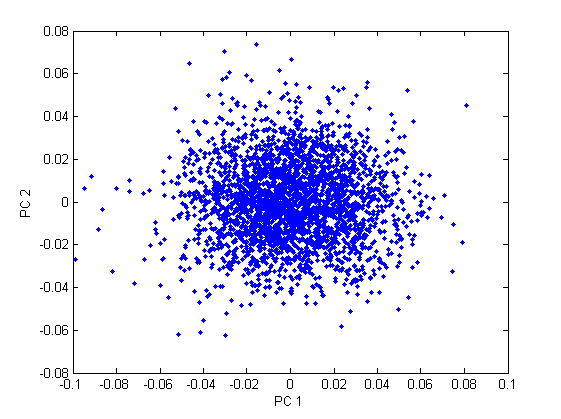
**Scatterplots of PCs:

**
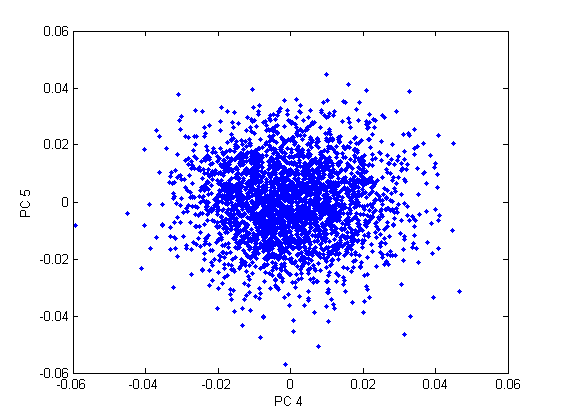

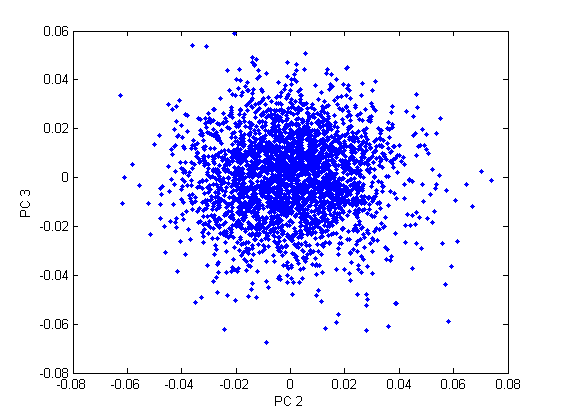
**

1. Facial morphs displaying the shape changes associated with each PC.


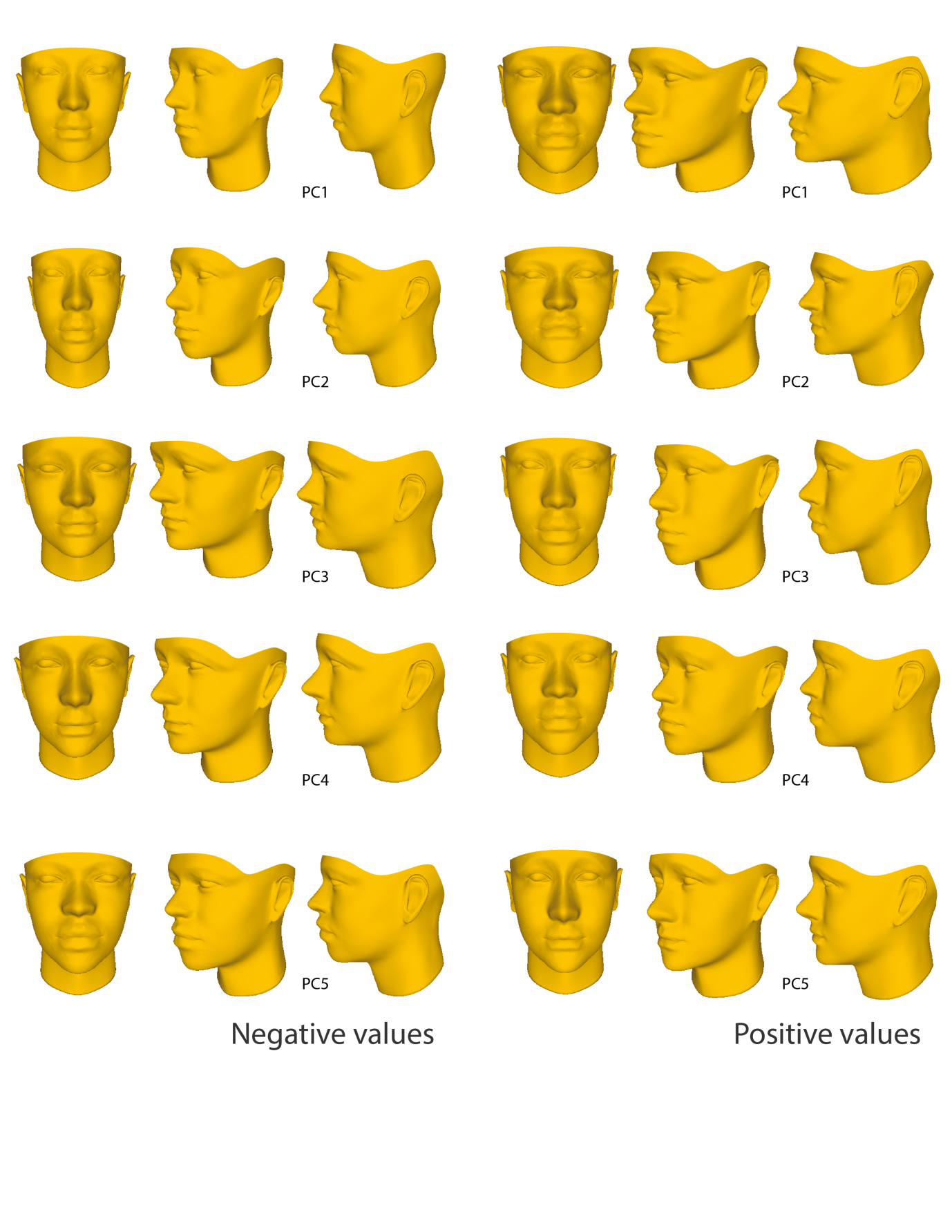

Supplement: Figure S3 — PCs 1–5 obtained from 3D coordinates of facial landmarks, represented by (A) Scatterplots and (B) Facial morphs. (DOCX) [file pgen.1004572.s003.docx]
