# Supplementary material for: Admixture in Latin America: Geographic Structure, Phenotypic Diversity and Self-Perception of Ancestry Based on 7,342 Individuals
Source: PLoS Genet. 2014 Sep 25;10(9):e1004572. doi: 10.1371/journal.pgen.1004572 (PMC4177621; doi:10.1371/journal.pgen.1004572)

**Supplementary Figure S4: Birthplace maps of study volunteers in (A) Brazil, (B) Chile, (C) Colombia, (D) México and (E) Perú.**

**Circle locations correspond to unique birthplaces. Circle sizes are proportional to the number of volunteers born at that location.**

**
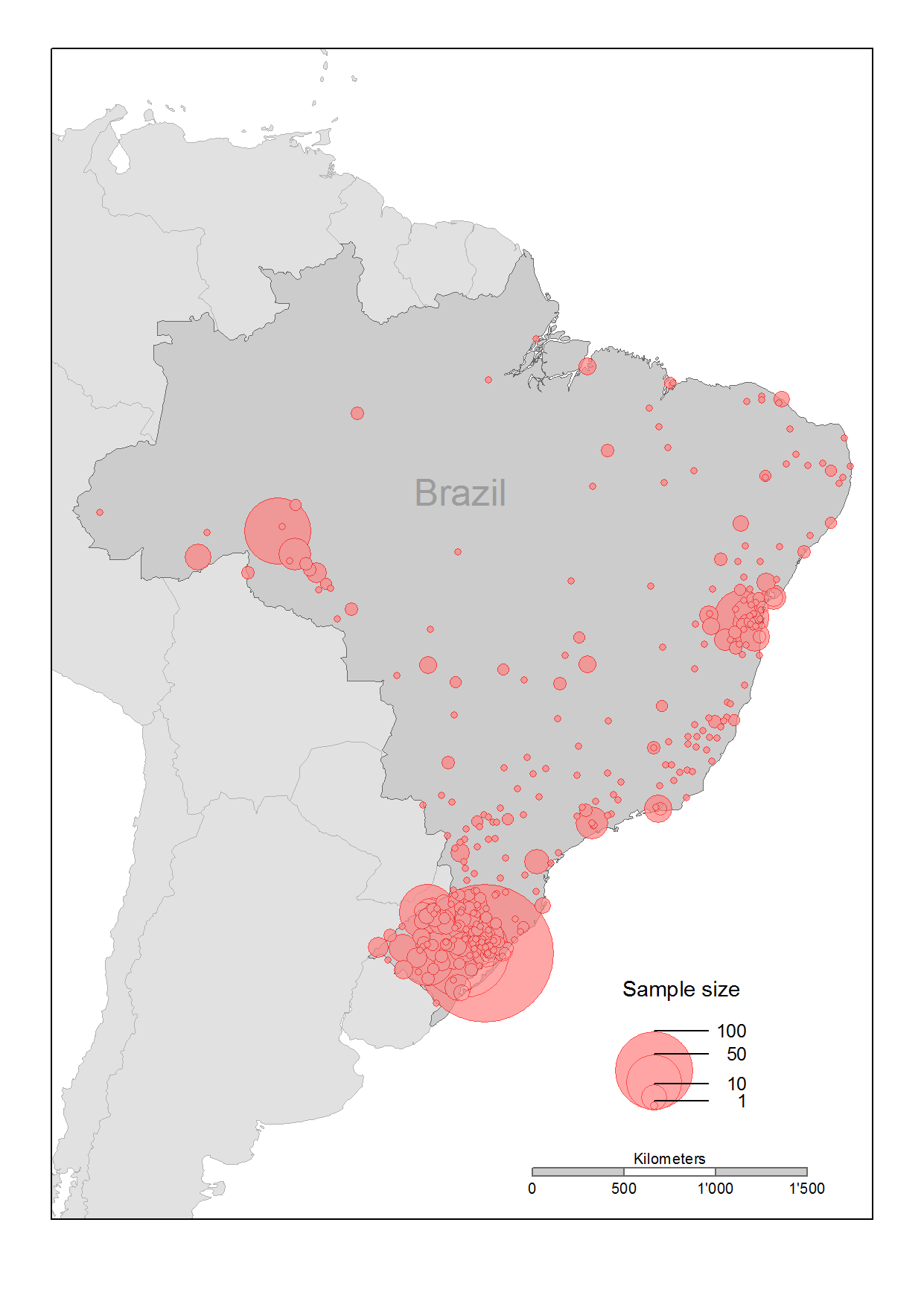
(A) Brazil**

**
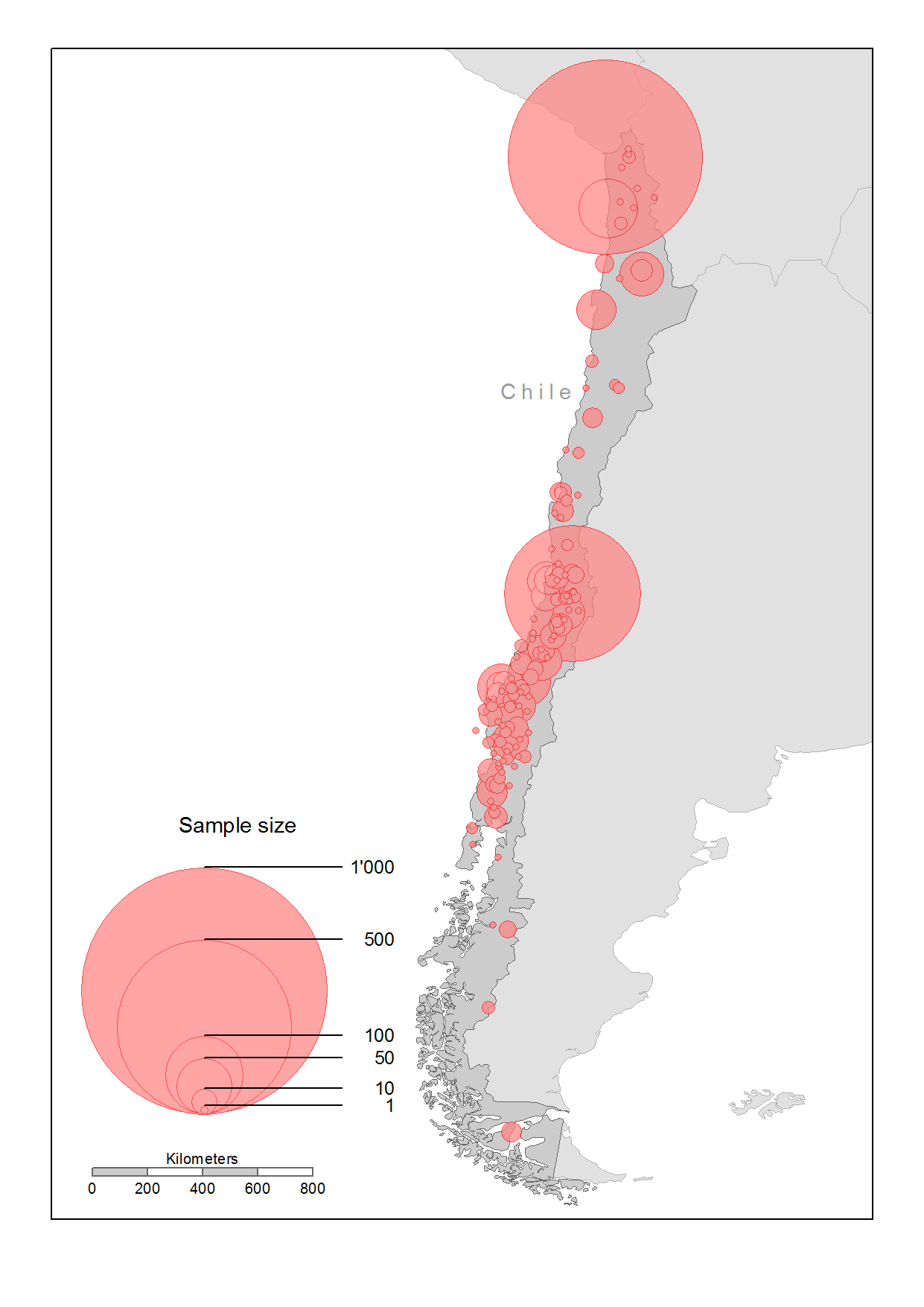
(B) Chile**

**(C) Colombia**

**
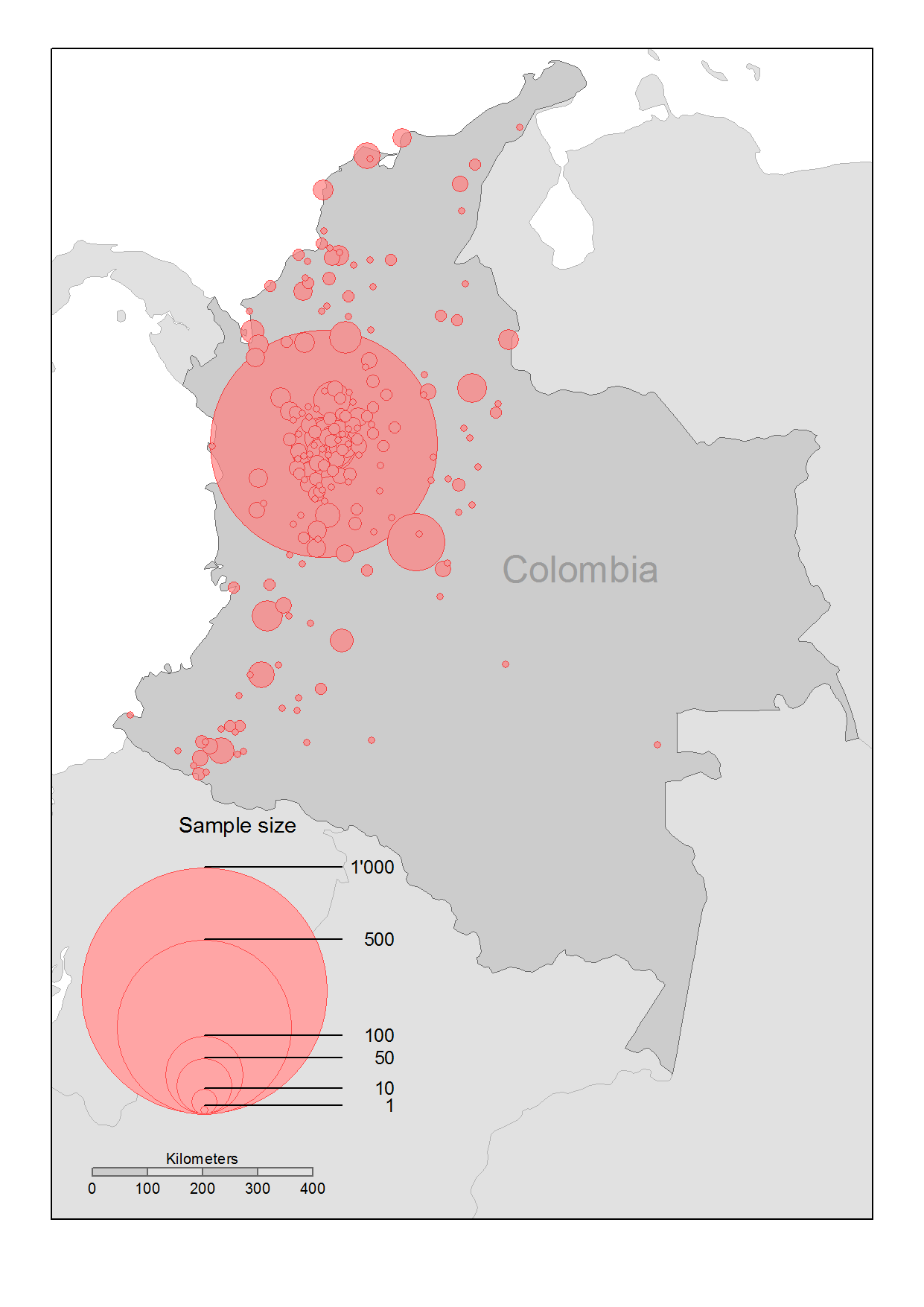
**

**(D) México**

**
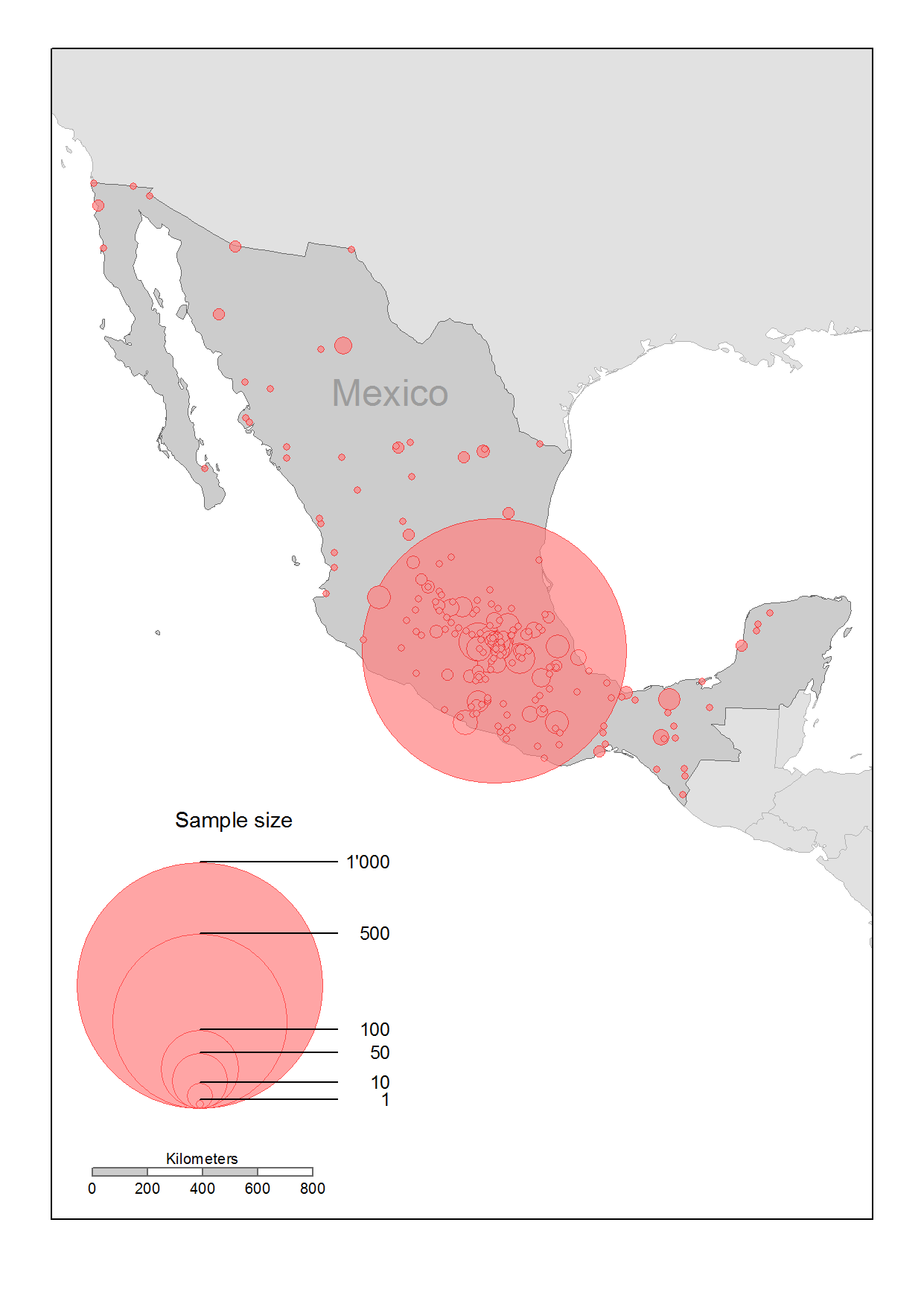
**

**(E) Perú**


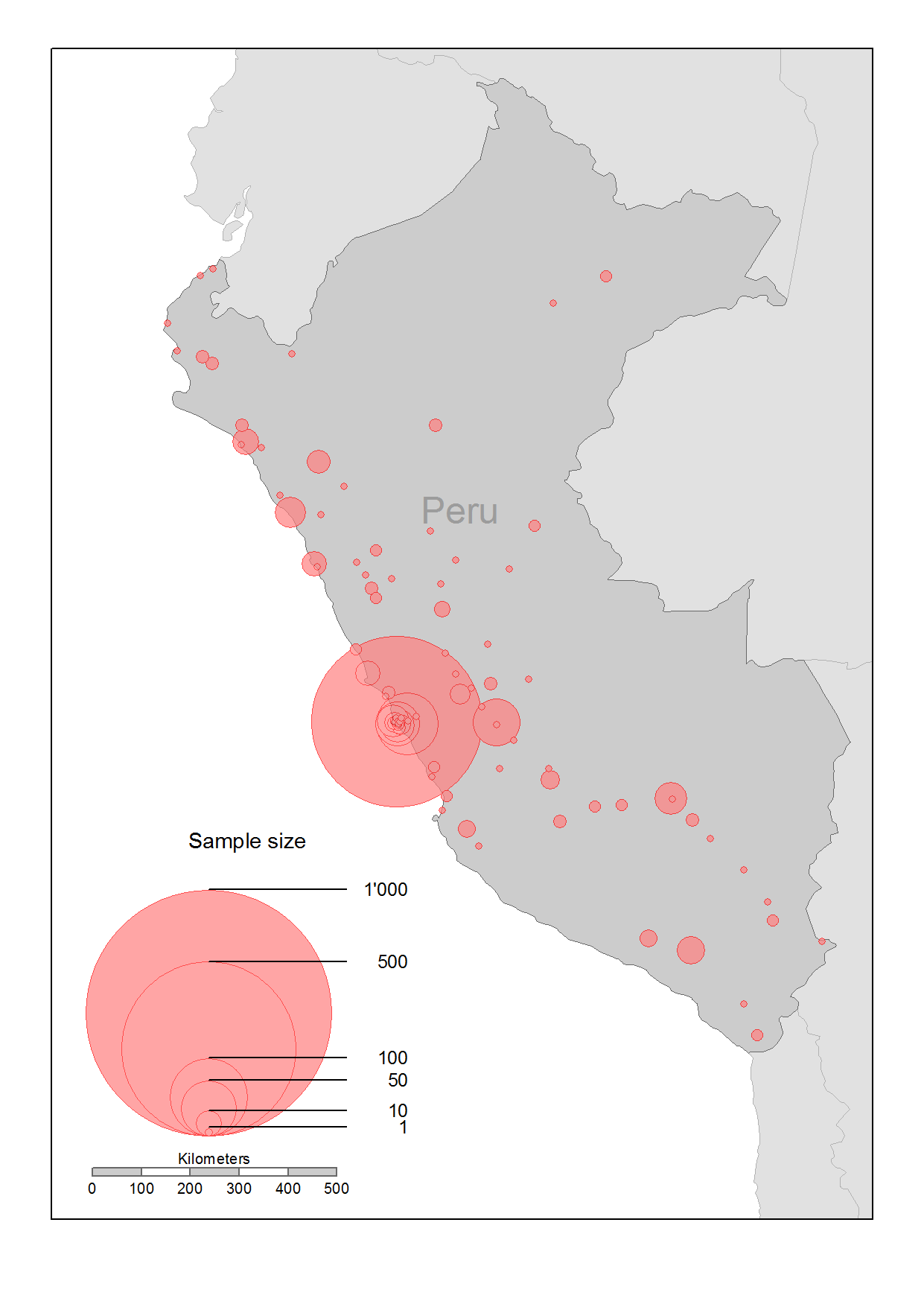

Supplement: Figure S4 — Birthplace maps of study volunteers in (A) Brazil, (B) Chile, (C) Colombia, (D) México and (E) Perú. (DOCX) [file pgen.1004572.s004.docx]
