# Supplementary material for: Admixture in Latin America: Geographic Structure, Phenotypic Diversity and Self-Perception of Ancestry Based on 7,342 Individuals
Source: PLoS Genet. 2014 Sep 25;10(9):e1004572. doi: 10.1371/journal.pgen.1004572 (PMC4177621; doi:10.1371/journal.pgen.1004572)

## Supplementary Figure S5: Populated locations (points) and Population sizes in (A) Brazil, (B) Chile, (C) Colombia, (D) México and (E) Perú.

Settlement point locations and population sizes were obtained from GRUMP ([http://sedac.ciesin.columbia.edu/data/set/grump-v1-settlement-points/](http://sedac.ciesin.columbia.edu/data/set/grump-v1-settlement-points/maps)). Population numbers from year 1990 are used, as the median age in our data ranges between 20-25 for each country.


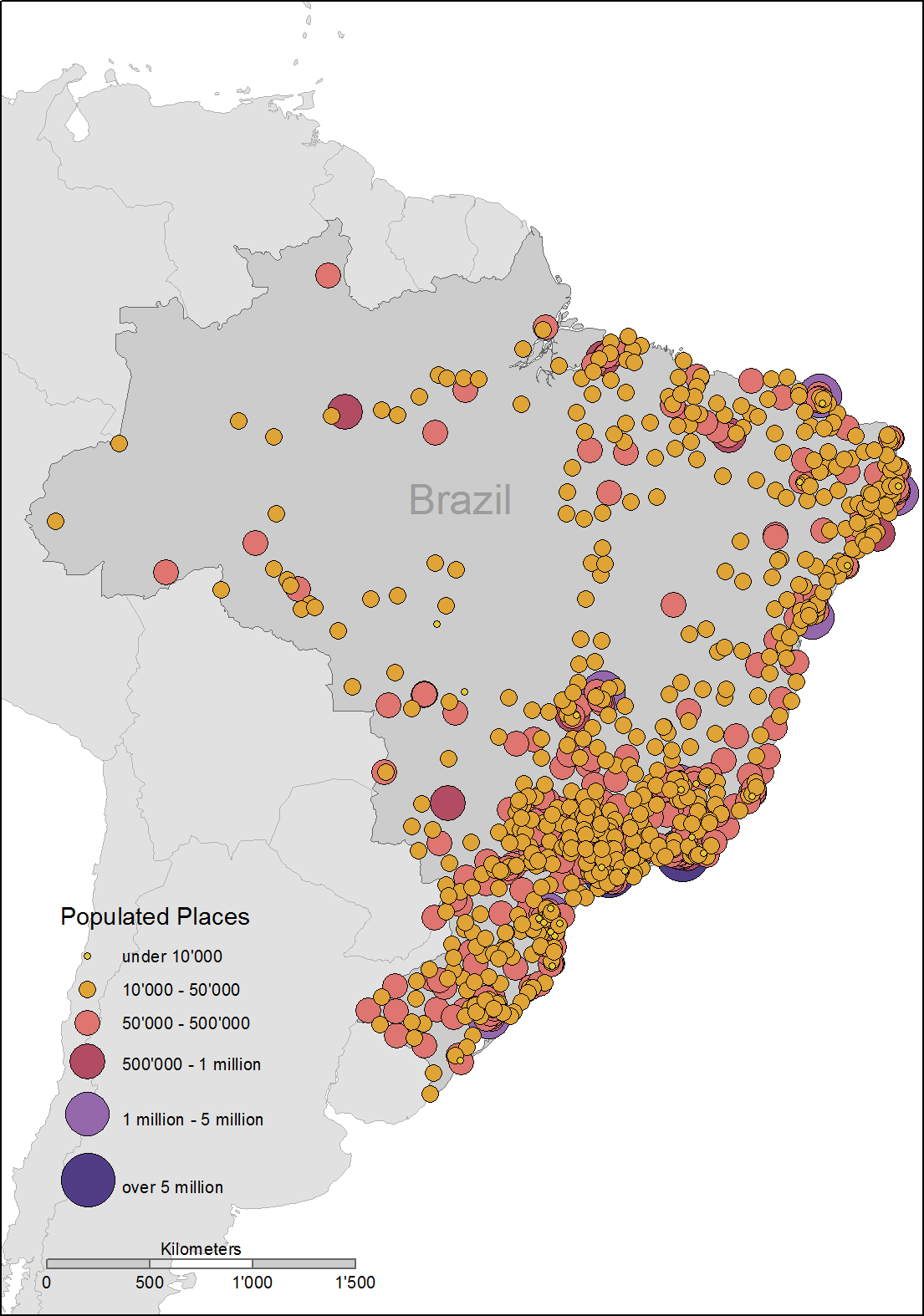
(A) Brazil

(B) Chile


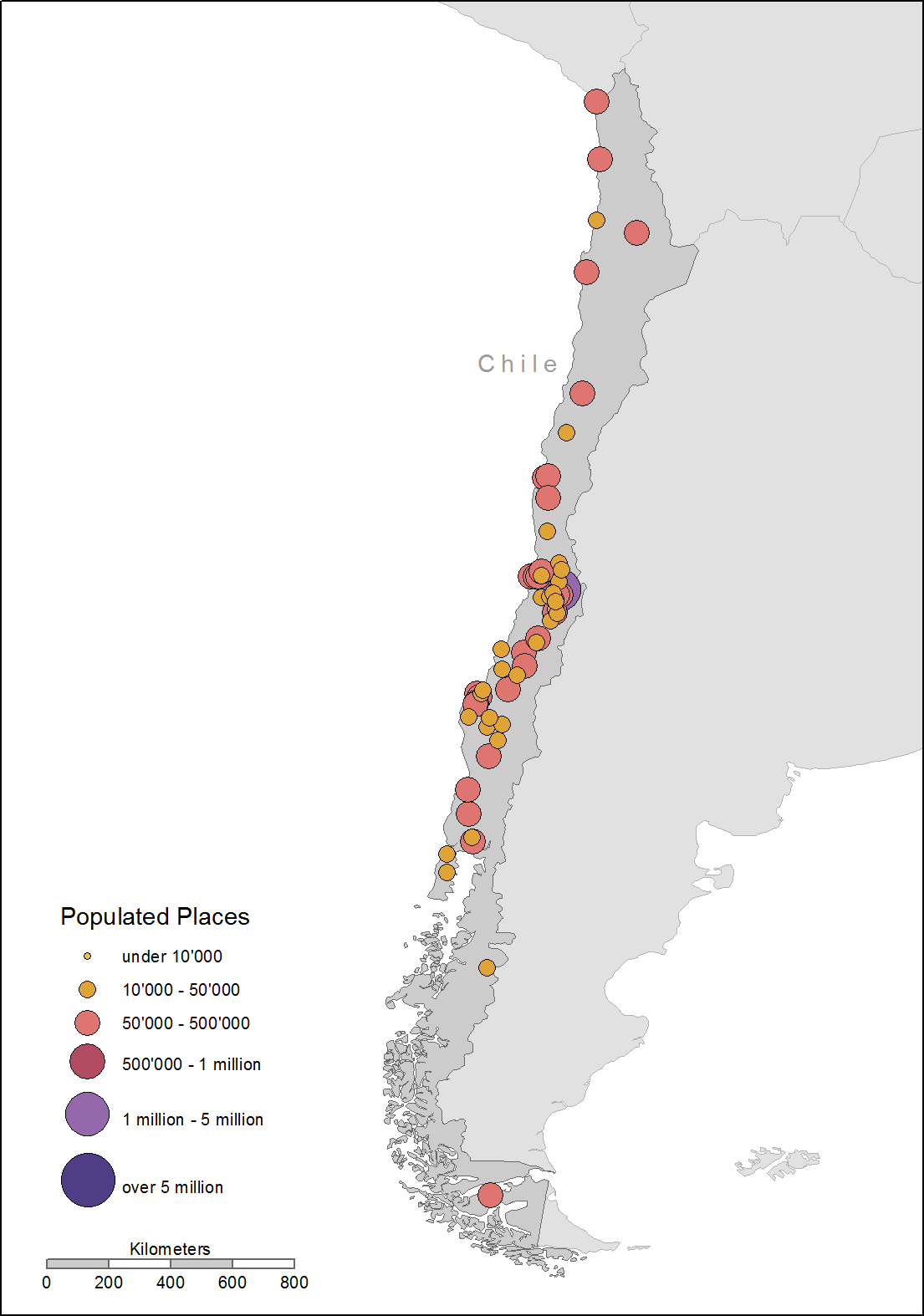


(C) Colombia


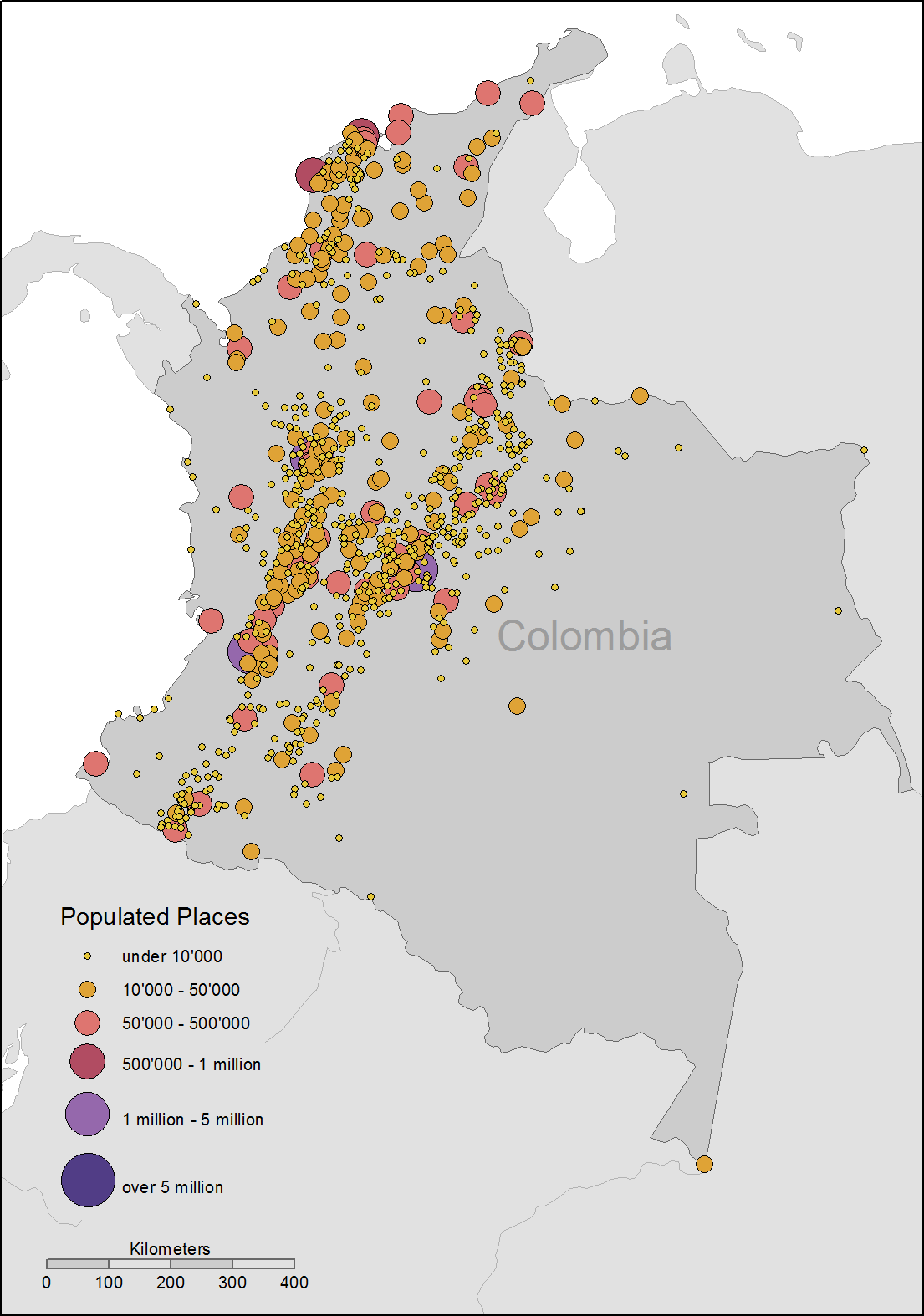


(D) Mexico


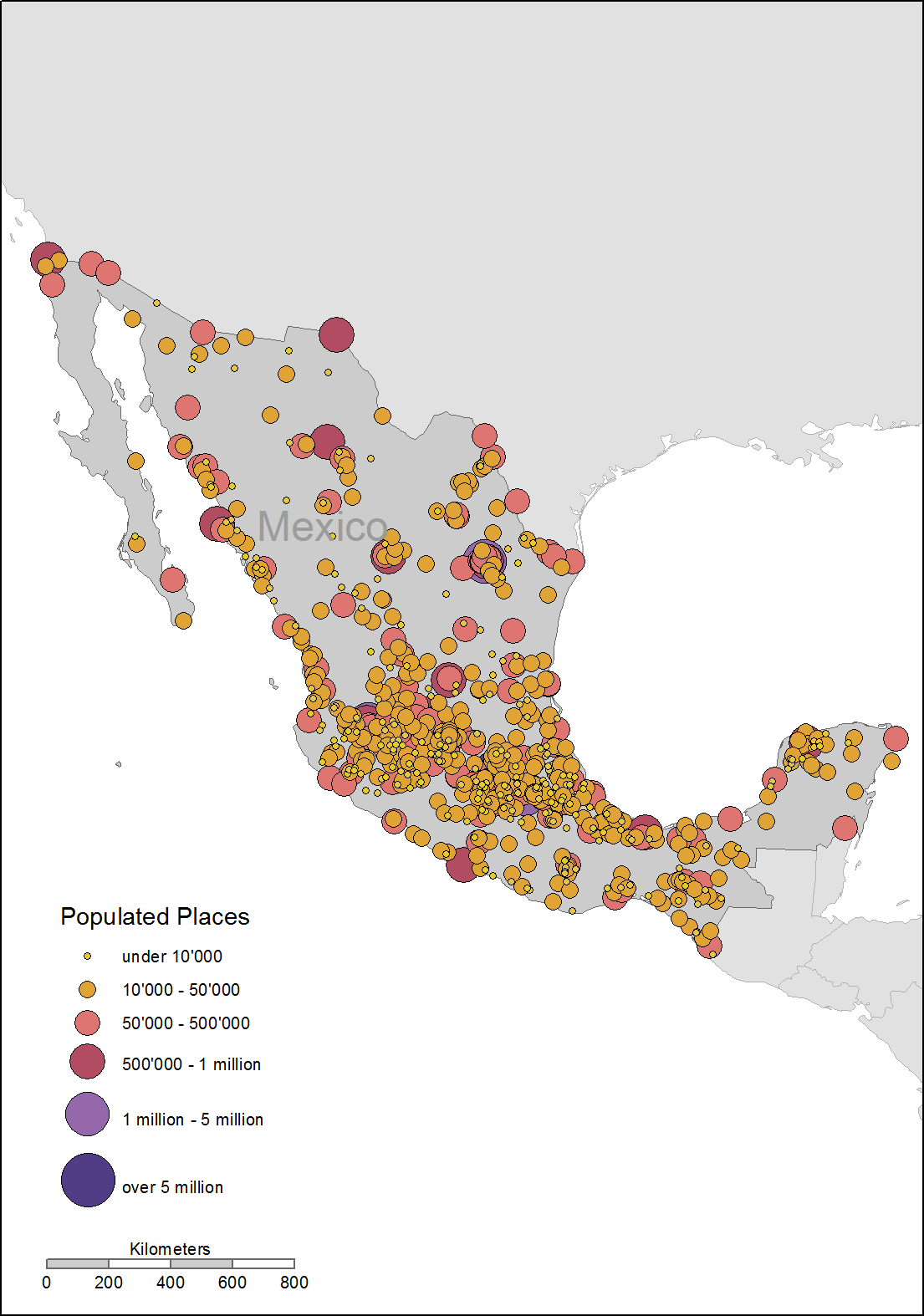


(E) Perú


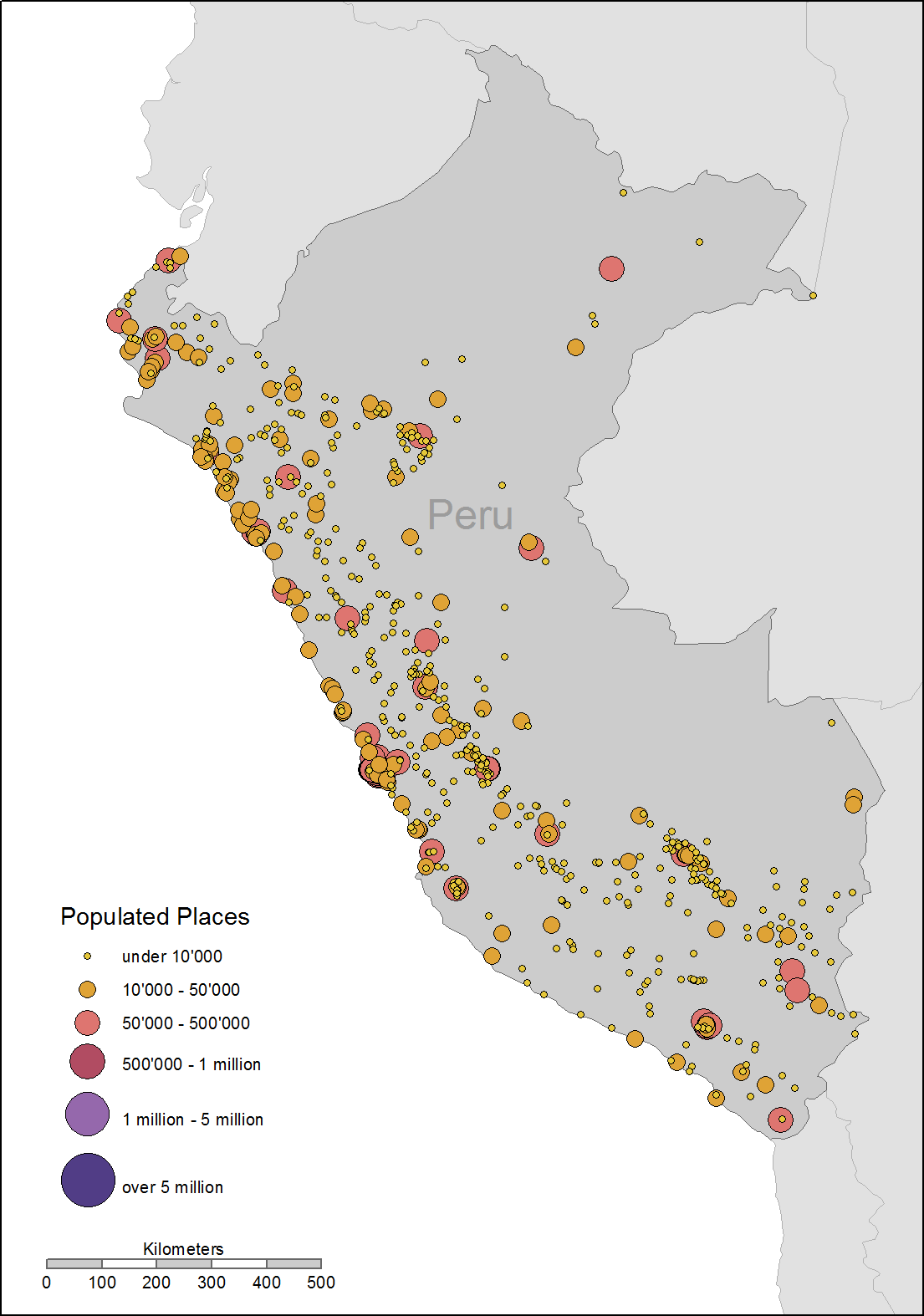

Supplement: Figure S5 — Populated locations (points) and Population sizes in (A) Brazil, (B) Chile, (C) Colombia, (D) México and (E) Perú. (DOCX) [file pgen.1004572.s005.docx]
