# Supplementary material for: Admixture in Latin America: Geographic Structure, Phenotypic Diversity and Self-Perception of Ancestry Based on 7,342 Individuals
Source: PLoS Genet. 2014 Sep 25;10(9):e1004572. doi: 10.1371/journal.pgen.1004572 (PMC4177621; doi:10.1371/journal.pgen.1004572)
Supplement: Figure S6 — Scatterplots of (A) Skin pigmentation (Melanin Index) and (B) Height (in cm), and European genetic ancestry. (DOCX) [file pgen.1004572.s006.docx]

**Supplementary Figure S6: Scatterplots of (A) Skin pigmentation (Melanin Index) and (B) Height (in cm), against European genetic ancestry.**

Linear fit obtained from regression analysis is shown as a black line.

1. Skin pigmentation (Melanin Index)


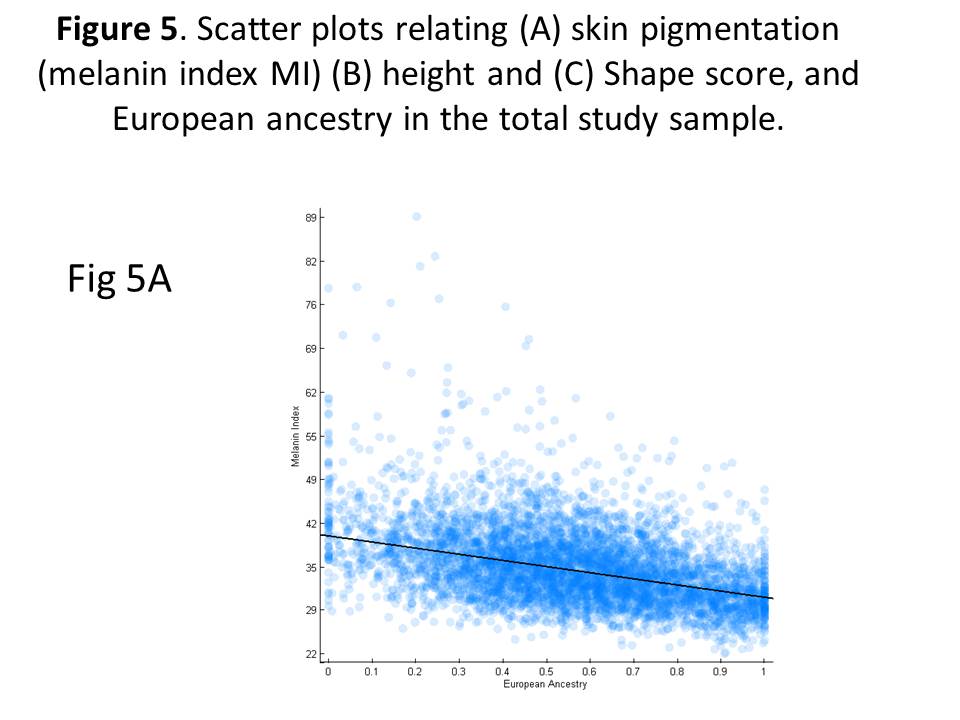


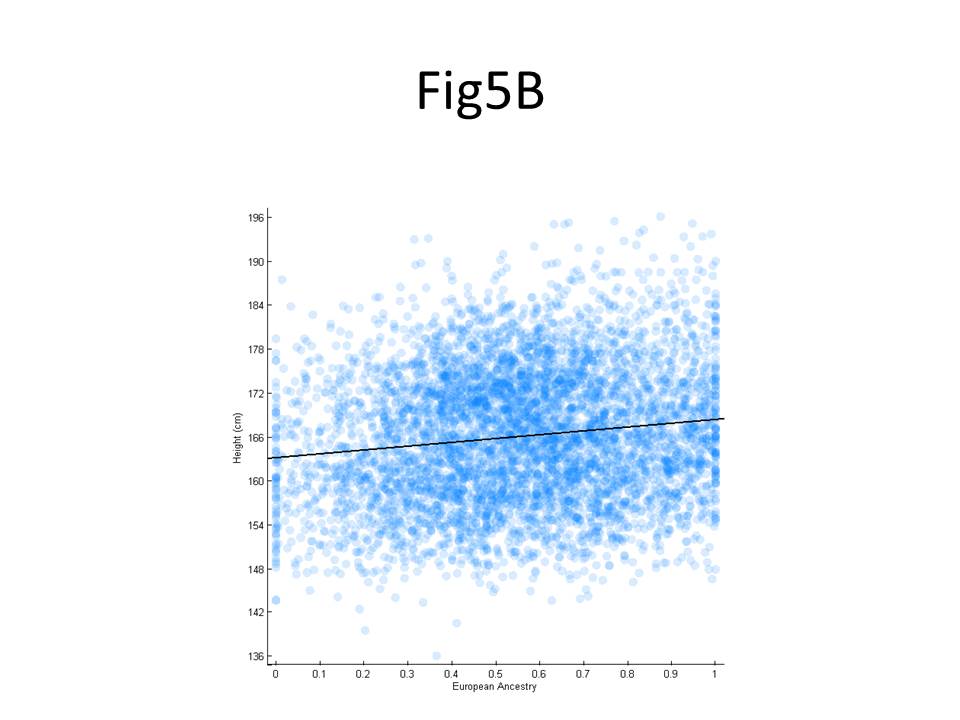


1. Height
