# Supplementary material for: Admixture in Latin America: Geographic Structure, Phenotypic Diversity and Self-Perception of Ancestry Based on 7,342 Individuals
Source: PLoS Genet. 2014 Sep 25;10(9):e1004572. doi: 10.1371/journal.pgen.1004572 (PMC4177621; doi:10.1371/journal.pgen.1004572)
Supplement: Table S1 — Allele frequencies at 30 SNP markers selected for African, European and Native American ancestry estimation and the absolute difference in reference allele frequency (Δ) between continental populations. (DOCX) [file pgen.1004572.s007.docx]

## Supplementary Table S1. Allele frequencies at 30 SNP markers selected for African, European and Native American ancestry estimation and the absolute difference in reference allele frequency (Δ) between continental populations.

|  |  |  |  | CONTINENTAL POPULATION | | | Δ | | |  |
| --- | --- | --- | --- | --- | --- | --- | --- | --- | --- | --- |
| SNP | C | Position | A1/A2 | AFRICA | AMERICA | EUROPE | Afr-Ame | Afr-Eur | Ame-Eur | Mean Δ |
| rs1544450 | 1 | 116,884,348 | G/T | 0.95 | 0.00 | 0.09 | 0.95 | 0.86 | 0.09 | 0.63 |
| rs1834619 | 2 | 17,901,485 | G/A | 0.00 | 0.97 | 0.04 | 0.97 | 0.04 | 0.94 | 0.65 |
| rs356652 | 2 | 101,540,415 | T/G | 0.00 | 0.93 | 0.07 | 0.93 | 0.07 | 0.87 | 0.62 |
| rs260690 | 2 | 109,579,738 | C/A | 0.64 | 0.96 | 0.05 | 0.32 | 0.60 | 0.92 | 0.61 |
| rs2176046 | 2 | 240,295,613 | G/A | 0.01 | 0.93 | 0.06 | 0.92 | 0.05 | 0.87 | 0.61 |
| rs10510511 | 3 | 21,260,370 | G/T | 0.00 | 0.92 | 0.02 | 0.92 | 0.02 | 0.89 | 0.61 |
| rs3870336 | 3 | 49,557,857 | G/A | 0.09 | 0.94 | 0.09 | 0.85 | 0.00 | 0.85 | 0.57 |
| rs10935320 | 3 | 139,056,584 | T/C | 0.15 | 0.98 | 0.10 | 0.83 | 0.05 | 0.88 | 0.58 |
| rs11725412 | 4 | 38,277,754 | A/G | 0.21 | 1.00 | 0.06 | 0.79 | 0.15 | 0.94 | 0.63 |
| rs10037656 | 5 | 5,183,783 | A/G | 0.34 | 0.98 | 0.10 | 0.64 | 0.24 | 0.88 | 0.59 |
| rs4145160 | 5 | 152,874,549 | G/A | 0.09 | 0.91 | 0.07 | 0.82 | 0.03 | 0.84 | 0.56 |
| rs1559163 | 5 | 154,911,447 | A/G | 0.00 | 0.85 | 0.02 | 0.85 | 0.02 | 0.83 | 0.57 |
| rs2042314 | 5 | 170,177,580 | C/T | 0.14 | 1.00 | 0.15 | 0.86 | 0.01 | 0.85 | 0.57 |
| rs12662498 | 6 | 56,188,650 | G/A | 0.01 | 0.98 | 0.07 | 0.97 | 0.06 | 0.91 | 0.65 |
| rs17086231 | 6 | 155,704,119 | C/T | 0.02 | 0.94 | 0.12 | 0.93 | 0.10 | 0.83 | 0.62 |
| rs6464749 | 7 | 146,020,936 | A/G | 0.89 | 0.00 | 0.05 | 0.89 | 0.84 | 0.05 | 0.60 |
| rs7018273 | 8 | 82,004,857 | A/G | 0.86 | 0.00 | 0.02 | 0.86 | 0.84 | 0.02 | 0.57 |
| rs12347078 | 9 | 344,508 | A/C | 0.88 | 0.00 | 0.04 | 0.88 | 0.84 | 0.04 | 0.58 |
| rs734241 | 10 | 115,239,602 | G/A | 0.04 | 0.99 | 0.07 | 0.94 | 0.02 | 0.92 | 0.63 |
| rs174570 | 11 | 61,597,212 | C/T | 0.01 | 1.00 | 0.11 | 0.99 | 0.10 | 0.89 | 0.66 |
| rs7134749 | 12 | 50,237,637 | T/C | 0.21 | 0.90 | 0.03 | 0.69 | 0.18 | 0.87 | 0.58 |
| rs2052386 | 12 | 68,876,835 | G/A | 0.08 | 0.93 | 0.10 | 0.85 | 0.02 | 0.83 | 0.57 |
| rs1849384 | 12 | 85,833,160 | A/C | 0.97 | 0.00 | 0.08 | 0.97 | 0.89 | 0.08 | 0.65 |
| rs4769128 | 13 | 21,322,682 | C/T | 0.15 | 0.99 | 0.13 | 0.83 | 0.02 | 0.86 | 0.57 |
| rs1243370 | 14 | 21,674,214 | T/C | 0.24 | 0.92 | 0.06 | 0.68 | 0.18 | 0.86 | 0.58 |
| rs2719921 | 15 | 25,491,630 | G/A | 0.88 | 0.00 | 0.03 | 0.88 | 0.84 | 0.03 | 0.58 |
| rs1197062 | 17 | 58,641,118 | T/G | 0.89 | 0.00 | 0.06 | 0.89 | 0.83 | 0.06 | 0.59 |
| rs717225 | 19 | 42,393,286 | A/G | 0.88 | 0.00 | 0.01 | 0.88 | 0.88 | 0.01 | 0.59 |
| rs6119879 | 20 | 31,105,389 | C/T | 0.69 | 0.00 | 0.84 | 0.69 | 0.15 | 0.84 | 0.56 |
| rs2426552 | 20 | 53,757,603 | C/T | 0.83 | 0.00 | 0.01 | 0.83 | 0.83 | 0.01 | 0.56 |

Positions of SNPS from GRCh37/HG19.
