# Supplementary material for: Admixture in Latin America: Geographic Structure, Phenotypic Diversity and Self-Perception of Ancestry Based on 7,342 Individuals
Source: PLoS Genet. 2014 Sep 25;10(9):e1004572. doi: 10.1371/journal.pgen.1004572 (PMC4177621; doi:10.1371/journal.pgen.1004572)
Supplement: Table S2 — Population samples used for genetic ancestry estimation. (DOCX) [file pgen.1004572.s008.docx]

## Supplementary Table S2: Population samples used for genetic ancestry estimation.

| AFRICA | N |  | EUROPE | N |  | AMERICA | N |
| --- | --- | --- | --- | --- | --- | --- | --- |
| YRI | 109 |  | CEU | 111 |  | PimaMX | 33 |
| Mandenka | 22 |  | TSI | 88 |  | Cabecar | 31 |
| Yoruba | 21 |  | French | 28 |  | Surui | 24 |
| BantuKenya | 9 |  | Sardinian | 28 |  | Aymara | 23 |
| BantuSouthAfrica | 8 |  | Basque | 24 |  | Zapotec | 23 |
| Total | 169 |  | Italian | 12 |  | PimaAZ | 22 |
|  |  |  | Tuscan | 8 |  | Quechua2 | 22 |
|  |  |  | Total | 299 |  | Maya | 18 |
|  |  |  |  |  |  | Quechua1 | 18 |
|  |  |  |  |  |  | Mixe | 17 |
|  |  |  |  |  |  | Kaqchikel | 13 |
|  |  |  |  |  |  | Karitiana | 13 |
|  |  |  |  |  |  | Ticuna2 | 12 |
|  |  |  |  |  |  | Wayuu | 12 |
|  |  |  |  |  |  | Inga | 10 |
|  |  |  |  |  |  | Chilote | 8 |
|  |  |  |  |  |  | Piapoco | 7 |
|  |  |  |  |  |  | Guahibo | 6 |
|  |  |  |  |  |  | Guarani | 6 |
|  |  |  |  |  |  | Ticuna1 | 6 |
|  |  |  |  |  |  | Arhuaco | 5 |
|  |  |  |  |  |  | Diaguita | 5 |
|  |  |  |  |  |  | Embera | 5 |
|  |  |  |  |  |  | Guaymi | 5 |
|  |  |  |  |  |  | Mixtec | 5 |
|  |  |  |  |  |  | Wichi | 5 |
|  |  |  |  |  |  | Zenu | 5 |
|  |  |  |  |  |  | Bribri | 4 |
|  |  |  |  |  |  | Chono | 4 |
|  |  |  |  |  |  | Hulliche | 4 |
|  |  |  |  |  |  | Kogi | 4 |
|  |  |  |  |  |  | Toba | 4 |
|  |  |  |  |  |  | Yaghan | 4 |
|  |  |  |  |  |  | Maleku | 3 |
|  |  |  |  |  |  | Palikur | 3 |
|  |  |  |  |  |  | Teribe | 3 |
|  |  |  |  |  |  | Waunana | 3 |
|  |  |  |  |  |  | Chane | 2 |
|  |  |  |  |  |  | Kaingang | 2 |
|  |  |  |  |  |  | Kalina | 2 |
|  |  |  |  |  |  | Arara | 1 |
|  |  |  |  |  |  | Chorotega | 1 |
|  |  |  |  |  |  | Huetar | 1 |
|  |  |  |  |  |  | Jamamadi | 1 |
|  |  |  |  |  |  | Parakana | 1 |
|  |  |  |  |  |  | Purepecha | 1 |
|  |  |  |  |  |  | Yaqui | 1 |
|  |  |  |  |  |  | Total | 408 |
