# Supplementary material for: Admixture in Latin America: Geographic Structure, Phenotypic Diversity and Self-Perception of Ancestry Based on 7,342 Individuals
Source: PLoS Genet. 2014 Sep 25;10(9):e1004572. doi: 10.1371/journal.pgen.1004572 (PMC4177621; doi:10.1371/journal.pgen.1004572)
Supplement: Table S3 — Coefficients and P-values for regression of pair-wise inter-landmark distances and ancestry. (DOCX) [file pgen.1004572.s009.docx]

## Supplementary Table S3: Coefficients and P-values for regression of pair-wise inter-landmark distances and ancestry.

Note: Only data for P-values<10^-6^ with European ancestry (133/427) are shown (ranked by European P-value). Landmarks are labelled as in Supplementary Figure S1. Equivalent distances between the two sides of the face have been averaged.

|  |  | African ancestry | | European Ancestry | |
| --- | --- | --- | --- | --- | --- |
| Distance # | Landmarks | Coef. | P-value | Coef. | P-value |
| 1 | 28.26.30 | 0.9 | 1.27E-02 | -1.9 | 2.00E-16 |
| 2 | 28.22.24 | 2.2 | 1.18E-03 | -3.1 | 2.58E-16 |
| 3 | 31.26.30 | 2.9 | 3.07E-05 | -3.2 | 3.13E-16 |
| 4 | 31.22.24 | 3.8 | 3.44E-05 | -4.0 | 2.34E-15 |
| 5 | 22.29.24.27 | 2.5 | 4.34E-03 | -3.8 | 4.49E-15 |
| 6 | 27.24.29.22 | 2.5 | 4.34E-03 | -3.8 | 4.49E-15 |
| 7 | 5.34.14.36 | -3.5 | 2.65E-07 | -2.9 | 1.11E-14 |
| 8 | 22.24 | 3.5 | 5.93E-06 | -3.2 | 1.10E-13 |
| 9 | 26.29.30.27 | 1.2 | 9.82E-02 | -3.0 | 1.59E-13 |
| 10 | 27.30.29.26 | 1.2 | 9.82E-02 | -3.0 | 1.59E-13 |
| 11 | 31.2.11 | 0.6 | 7.31E-01 | -7.5 | 6.32E-13 |
| 12 | 25.28 | 1.0 | 4.80E-03 | -1.4 | 4.27E-12 |
| 13 | 25.31 | 3.1 | 1.08E-05 | -2.7 | 6.38E-12 |
| 14 | 25.27.29 | 1.3 | 4.86E-02 | -2.4 | 7.69E-12 |
| 15 | 28.2.11 | -1.1 | 5.22E-01 | -6.5 | 7.78E-12 |
| 16 | 5.36.14.34 | -4.6 | 4.31E-02 | -8.5 | 8.89E-12 |
| 17 | 34.14.36.5 | -4.6 | 4.31E-02 | -8.5 | 8.89E-12 |
| 18 | 32.26.30 | 4.0 | 5.55E-04 | -4.3 | 9.81E-12 |
| 19 | 2.29.11.27 | -0.8 | 6.56E-01 | -6.8 | 9.89E-12 |
| 20 | 27.11.29.2 | -0.8 | 6.56E-01 | -6.8 | 9.89E-12 |
| 21 | 31.3.12 | 0.6 | 7.31E-01 | -6.9 | 1.31E-11 |
| 22 | 34.36 | -3.1 | 1.73E-01 | -8.4 | 1.60E-11 |
| 23 | 23.22.24 | 1.3 | 4.73E-03 | -1.7 | 1.73E-11 |
| 24 | 3.29.12.27 | -1.2 | 4.90E-01 | -6.7 | 1.88E-11 |
| 25 | 27.12.29.3 | -1.2 | 4.90E-01 | -6.7 | 1.88E-11 |
| 26 | 31.10.19 | 0.3 | 8.50E-01 | -5.2 | 2.08E-11 |
| 27 | 10.29.19.27 | -0.6 | 6.33E-01 | -4.8 | 2.96E-11 |
| 28 | 27.19.29.10 | -0.6 | 6.33E-01 | -4.8 | 2.96E-11 |
| 29 | 22.30.24.26 | 2.1 | 2.16E-03 | -2.5 | 5.31E-11 |
| 30 | 26.24.30.22 | 2.1 | 2.16E-03 | -2.5 | 5.31E-11 |
| 31 | 25.22.24 | 1.9 | 4.22E-03 | -2.4 | 5.46E-11 |
| 32 | 27.29 | 1.0 | 2.66E-01 | -3.3 | 9.35E-11 |
| 33 | 26.27.30.29 | 1.3 | 3.71E-02 | -2.1 | 1.27E-10 |
| 34 | 31.7.16 | 1.2 | 4.42E-01 | -5.4 | 1.45E-10 |
| 35 | 3.36.12.34 | -3.7 | 1.13E-01 | -8.1 | 2.00E-10 |
| 36 | 34.12.36.3 | -3.7 | 1.13E-01 | -8.1 | 2.00E-10 |
| 37 | 2.27.11.29 | -1.6 | 3.29E-01 | -5.8 | 2.01E-10 |
| 38 | 28.3.12 | -0.9 | 5.98E-01 | -6.0 | 2.02E-10 |
| 39 | 31.27.29 | 1.4 | 2.62E-02 | -2.2 | 2.17E-10 |
| 40 | 2.24.11.22 | -0.4 | 7.98E-01 | -5.1 | 2.26E-10 |
| 41 | 22.11.24.2 | -0.4 | 7.98E-01 | -5.1 | 2.26E-10 |
| 42 | 33.36.35.34 | -6.4 | 5.97E-03 | -8.2 | 2.43E-10 |
| 43 | 34.35.36.33 | -6.4 | 5.97E-03 | -8.2 | 2.43E-10 |
| 44 | 28.10.19 | -1.3 | 2.83E-01 | -4.2 | 3.18E-10 |
| 45 | 25.32 | 4.2 | 2.30E-04 | -3.9 | 3.53E-10 |
| 46 | 7.29.16.27 | 0.1 | 9.70E-01 | -5.0 | 5.81E-10 |
| 47 | 27.16.29.7 | 0.1 | 9.70E-01 | -5.0 | 5.81E-10 |
| 48 | 20.31 | 0.2 | 8.90E-01 | -5.1 | 6.17E-10 |
| 49 | 23.31 | 2.9 | 1.13E-03 | -3.0 | 6.61E-10 |
| 50 | 3.27.12.29 | -1.4 | 3.68E-01 | -5.5 | 7.65E-10 |
| 51 | 5.14 | -5.1 | 2.69E-02 | -7.8 | 8.80E-10 |
| 52 | 2.8.11.17 | 0.6 | 4.17E-01 | -2.4 | 8.93E-10 |
| 53 | 28.27.29 | 0.9 | 1.44E-01 | -1.9 | 1.43E-09 |
| 54 | 32.2.11 | 0.4 | 8.47E-01 | -7.5 | 1.66E-09 |
| 55 | 9.29.18.27 | -1.0 | 4.13E-01 | -4.0 | 2.06E-09 |
| 56 | 27.18.29.9 | -1.0 | 4.13E-01 | -4.0 | 2.06E-09 |
| 57 | 6.36.15.34 | -2.0 | 3.94E-01 | -7.6 | 2.28E-09 |
| 58 | 34.15.36.6 | -2.0 | 3.94E-01 | -7.6 | 2.28E-09 |
| 59 | 28.7.16 | -0.3 | 8.45E-01 | -4.4 | 2.31E-09 |
| 60 | 10.27.19.29 | -2.5 | 2.80E-02 | -3.7 | 2.42E-09 |
| 61 | 31.8.17 | 0.0 | 9.76E-01 | -5.0 | 2.89E-09 |
| 62 | 5.15.14.6 | -3.4 | 1.44E-01 | -7.6 | 3.29E-09 |
| 63 | 6.14.15.5 | -3.4 | 1.44E-01 | -7.6 | 3.29E-09 |
| 64 | 5.6.14.15 | -2.3 | 2.88E-04 | -2.1 | 3.36E-09 |
| 65 | 32.3.12 | -0.1 | 9.59E-01 | -7.1 | 4.43E-09 |
| 66 | 8.29.17.27 | -1.0 | 4.58E-01 | -4.6 | 4.59E-09 |
| 67 | 27.17.29.8 | -1.0 | 4.58E-01 | -4.6 | 4.59E-09 |
| 68 | 4.36.13.34 | -5.7 | 2.15E-02 | -8.0 | 4.95E-09 |
| 69 | 34.13.36.4 | -5.7 | 2.15E-02 | -8.0 | 4.95E-09 |
| 70 | 31.9.18 | 0.7 | 5.91E-01 | -4.4 | 5.13E-09 |
| 71 | 10.24.19.22 | 0.8 | 4.35E-01 | -3.2 | 5.66E-09 |
| 72 | 22.19.24.10 | 0.8 | 4.35E-01 | -3.2 | 5.66E-09 |
| 73 | 3.34.12.36 | -1.9 | 2.75E-01 | -5.5 | 6.27E-09 |
| 74 | 25.2.11 | -1.9 | 2.59E-01 | -5.2 | 1.53E-08 |
| 75 | 23.27.29 | 0.5 | 5.01E-01 | -2.1 | 1.94E-08 |
| 76 | 23.2.11 | -2.1 | 1.53E-01 | -4.5 | 2.24E-08 |
| 77 | 20.28 | -1.8 | 1.66E-01 | -4.0 | 2.26E-08 |
| 78 | 25.26.30 | 0.1 | 7.34E-01 | -0.8 | 2.51E-08 |
| 79 | 3.24.12.22 | -0.8 | 6.16E-01 | -4.6 | 2.91E-08 |
| 80 | 22.12.24.3 | -0.8 | 6.16E-01 | -4.6 | 2.91E-08 |
| 81 | 33.34.35.36 | -5.5 | 1.11E-07 | -3.1 | 3.13E-08 |
| 82 | 5.35.14.33 | -6.3 | 7.54E-03 | -7.1 | 3.36E-08 |
| 83 | 33.14.35.5 | -6.3 | 7.54E-03 | -7.1 | 3.36E-08 |
| 84 | 7.27.16.29 | -1.4 | 2.54E-01 | -3.8 | 3.44E-08 |
| 85 | 1.31 | 1.4 | 4.18E-01 | -5.2 | 4.20E-08 |
| 86 | 2.30.11.26 | -1.7 | 3.12E-01 | -5.1 | 4.68E-08 |
| 87 | 26.11.30.2 | -1.7 | 3.12E-01 | -5.1 | 4.68E-08 |
| 88 | 28.8.17 | -1.8 | 1.87E-01 | -4.0 | 5.98E-08 |
| 89 | 32.10.19 | -0.3 | 8.73E-01 | -5.3 | 6.58E-08 |
| 90 | 6.35.15.33 | -5.1 | 3.33E-02 | -7.1 | 6.79E-08 |
| 91 | 33.15.35.6 | -5.1 | 3.33E-02 | -7.1 | 6.79E-08 |
| 92 | 6.34.15.36 | 2.8 | 4.44E-06 | 1.8 | 7.73E-08 |
| 93 | 2.9.11.18 | 0.3 | 7.48E-01 | -2.3 | 9.34E-08 |
| 94 | 3.14.12.5 | -2.9 | 2.02E-01 | -6.7 | 9.62E-08 |
| 95 | 5.12.14.3 | -2.9 | 2.02E-01 | -6.7 | 9.62E-08 |
| 96 | 28.9.18 | -1.1 | 3.70E-01 | -3.4 | 1.01E-07 |
| 97 | 20.27.29 | -1.8 | 1.74E-01 | -3.8 | 1.02E-07 |
| 98 | 2.18.11.9 | 0.3 | 8.14E-01 | -3.5 | 1.16E-07 |
| 99 | 9.11.18.2 | 0.3 | 8.14E-01 | -3.5 | 1.16E-07 |
| 100 | 22.27.24.29 | 0.7 | 2.71E-01 | -1.9 | 1.46E-07 |
| 101 | 6.15 | -1.1 | 6.47E-01 | -6.9 | 1.58E-07 |
| 102 | 32.22.24 | 3.6 | 8.78E-03 | -3.9 | 1.63E-07 |
| 103 | 2.22.11.24 | -3.0 | 1.50E-02 | -3.5 | 1.72E-07 |
| 104 | 32.7.16 | 0.4 | 8.46E-01 | -5.3 | 2.41E-07 |
| 105 | 7.24.16.22 | 1.2 | 3.15E-01 | -3.3 | 2.73E-07 |
| 106 | 22.16.24.7 | 1.2 | 3.15E-01 | -3.3 | 2.73E-07 |
| 107 | 5.29.14.27 | 1.9 | 3.61E-01 | -5.8 | 2.98E-07 |
| 108 | 27.14.29.5 | 1.9 | 3.61E-01 | -5.8 | 2.98E-07 |
| 109 | 8.24.17.22 | -0.1 | 9.09E-01 | -3.0 | 3.00E-07 |
| 110 | 22.17.24.8 | -0.1 | 9.09E-01 | -3.0 | 3.00E-07 |
| 111 | 23.28 | 0.7 | 2.55E-01 | -1.7 | 3.50E-07 |
| 112 | 23.32 | 3.3 | 1.48E-02 | -3.8 | 3.61E-07 |
| 113 | 8.27.17.29 | -2.5 | 5.29E-02 | -3.6 | 3.81E-07 |
| 114 | 9.27.18.29 | -1.5 | 1.99E-01 | -3.2 | 3.82E-07 |
| 115 | 7.36.16.34 | -0.7 | 7.46E-01 | -5.8 | 3.99E-07 |
| 116 | 34.16.36.7 | -0.7 | 7.46E-01 | -5.8 | 3.99E-07 |
| 117 | 25.3.12 | -1.4 | 3.93E-01 | -4.7 | 4.11E-07 |
| 118 | 9.36.18.34 | -0.9 | 6.38E-01 | -5.2 | 4.46E-07 |
| 119 | 34.18.36.9 | -0.9 | 6.38E-01 | -5.2 | 4.46E-07 |
| 120 | 2.26.11.30 | -2.0 | 2.26E-01 | -4.5 | 4.70E-07 |
| 121 | 4.15.13.6 | -4.5 | 7.71E-02 | -7.1 | 5.27E-07 |
| 122 | 6.13.15.4 | -4.5 | 7.71E-02 | -7.1 | 5.27E-07 |
| 123 | 10.36.19.34 | 0.0 | 9.92E-01 | -5.6 | 5.63E-07 |
| 124 | 34.19.36.10 | 0.0 | 9.92E-01 | -5.6 | 5.63E-07 |
| 125 | 3.30.12.26 | -1.4 | 4.02E-01 | -4.6 | 5.96E-07 |
| 126 | 26.12.30.3 | -1.4 | 4.02E-01 | -4.6 | 5.96E-07 |
| 127 | 4.14.13.5 | -5.5 | 2.46E-02 | -6.8 | 5.99E-07 |
| 128 | 5.13.14.4 | -5.5 | 2.46E-02 | -6.8 | 5.99E-07 |
| 129 | 20.32 | 0.2 | 9.14E-01 | -5.3 | 7.63E-07 |
| 130 | 32.8.17 | -0.2 | 8.98E-01 | -5.2 | 8.08E-07 |
| 131 | 4.34.13.36 | -4.6 | 8.47E-05 | -3.2 | 8.35E-07 |
| 132 | 3.15.12.6 | -1.3 | 5.78E-01 | -6.5 | 9.32E-07 |
| 133 | 6.12.15.3 | -1.3 | 5.78E-01 | -6.5 | 9.32E-07 |
