# Supplementary material for: Admixture in Latin America: Geographic Structure, Phenotypic Diversity and Self-Perception of Ancestry Based on 7,342 Individuals
Source: PLoS Genet. 2014 Sep 25;10(9):e1004572. doi: 10.1371/journal.pgen.1004572 (PMC4177621; doi:10.1371/journal.pgen.1004572)
Supplement: Table S4 — Frequency (and %) of four ethnicity categories in each country, mean individual genetic ancestry and Melanin Index in each category. (DOCX) [file pgen.1004572.s010.docx]

## Supplementary Table S4. Frequency (and %) of four ethnicity categories in each country, mean individual genetic ancestry and Melanin Index in each category.

| \| COUNTRY \| Ethnicity \| % \| No. \| African \| American \| European \| MEI \| \| --- \| --- \| --- \| --- \| --- \| --- \| --- \| --- \| \| BRAZIL \| Black \| 5.8% \| 79 \| 0.49 \| 0.11 \| 0.39 \| 50.6 \| \|  \| White \| 72.7% \| 993 \| 0.08 \| 0.07 \| 0.85 \| 30.7 \| \|  \| Native \| 1.0% \| 13 \| 0.23 \| 0.17 \| 0.61 \| 38.5 \| \|  \| Mixed \| 20.5% \| 280 \| 0.18 \| 0.13 \| 0.69 \| 36.0 \| \| CHILE \| Black \| 0.9% \| 9 \| 0.13 \| 0.48 \| 0.39 \| 38.7 \| \|  \| White \| 37.9% \| 395 \| 0.05 \| 0.40 \| 0.54 \| 32.2 \| \|  \| Native \| 9.0% \| 94 \| 0.03 \| 0.76 \| 0.22 \| 40.6 \| \|  \| Mixed \| 52.2% \| 544 \| 0.05 \| 0.47 \| 0.49 \| 34.8 \| \| COLOMBIA \| Black \| 1.7% \| 23 \| 0.69 \| 0.12 \| 0.19 \| 64.1 \| \|  \| White \| 19.3% \| 258 \| 0.09 \| 0.26 \| 0.65 \| 30.5 \| \|  \| Native \| 2.0% \| 27 \| 0.12 \| 0.42 \| 0.46 \| 40.0 \| \|  \| Mixed \| 77.0% \| 1029 \| 0.11 \| 0.29 \| 0.60 \| 34.4 \| \| MEXICO \| Black \| 0.3% \| 3 \| 0.37 \| 0.41 \| 0.22 \| 48.2 \| \|  \| White \| 14.6% \| 164 \| 0.05 \| 0.42 \| 0.53 \| 31.5 \| \|  \| Native \| 4.8% \| 54 \| 0.03 \| 0.77 \| 0.20 \| 40.3 \| \|  \| Mixed \| 80.4% \| 905 \| 0.05 \| 0.56 \| 0.39 \| 35.5 \| \| PERU \| Black \| 0.0% \| 0 \| NA \| NA \| NA \| NA \| \|  \| White \| 11.3% \| 42 \| 0.05 \| 0.48 \| 0.47 \| 32.2 \| \|  \| Native \| 1.6% \| 6 \| 0.01 \| 0.84 \| 0.14 \| 40.4 \| \|  \| Mixed \| 87.1% \| 325 \| 0.05 \| 0.65 \| 0.30 \| 37.0 \| |
| --- | --- | --- | --- | --- | --- | --- | --- | --- | --- | --- | --- | --- | --- | --- | --- | --- | --- | --- | --- | --- | --- | --- | --- | --- | --- | --- | --- | --- | --- | --- | --- | --- | --- | --- | --- | --- | --- | --- | --- | --- | --- | --- | --- | --- | --- | --- | --- | --- | --- | --- | --- | --- | --- | --- | --- | --- | --- | --- | --- | --- | --- | --- | --- | --- | --- | --- | --- | --- | --- | --- | --- | --- | --- | --- | --- | --- | --- | --- | --- | --- | --- | --- | --- | --- | --- | --- | --- | --- | --- | --- | --- | --- | --- | --- | --- | --- | --- | --- | --- | --- | --- | --- | --- | --- | --- | --- | --- | --- | --- | --- | --- | --- | --- | --- | --- | --- | --- | --- | --- | --- | --- | --- | --- | --- | --- | --- | --- | --- | --- | --- | --- | --- | --- | --- | --- | --- | --- | --- | --- | --- | --- | --- | --- | --- | --- | --- | --- | --- | --- | --- | --- | --- | --- | --- | --- | --- | --- | --- | --- | --- | --- | --- | --- | --- | --- | --- | --- | --- |
