# Supplementary material for: Admixture in Latin America: Geographic Structure, Phenotypic Diversity and Self-Perception of Ancestry Based on 7,342 Individuals
Source: PLoS Genet. 2014 Sep 25;10(9):e1004572. doi: 10.1371/journal.pgen.1004572 (PMC4177621; doi:10.1371/journal.pgen.1004572)
Supplement: Text S1 — Variation in ancestry across countries and individuals. (DOCX) [file pgen.1004572.s011.docx]

## Supplementary Text S1: Variation in ancestry across countries and individuals

Throughout this paper we use the terms ‘African(s)’, ‘European(s)’ and ‘Native American(s)’ to refer broadly to different geographic origins without assuming a biological homogeneity, or strict distinctiveness, of these regions. Our main aim here is to obtain continental ancestry estimates consistently across samples as an heuristic allowing us to examine patterns of variation in continental ancestry in relation to geography, phenotype and self-perception. In this paper we use the country names simply as labels to indicate where recruitment took place. Below we present trivariate histograms for African, Native American and European ancestry for each country sample. The base is restricted to an equilateral triangle due to the constraint that the ancestry proportions sum up to 100%.


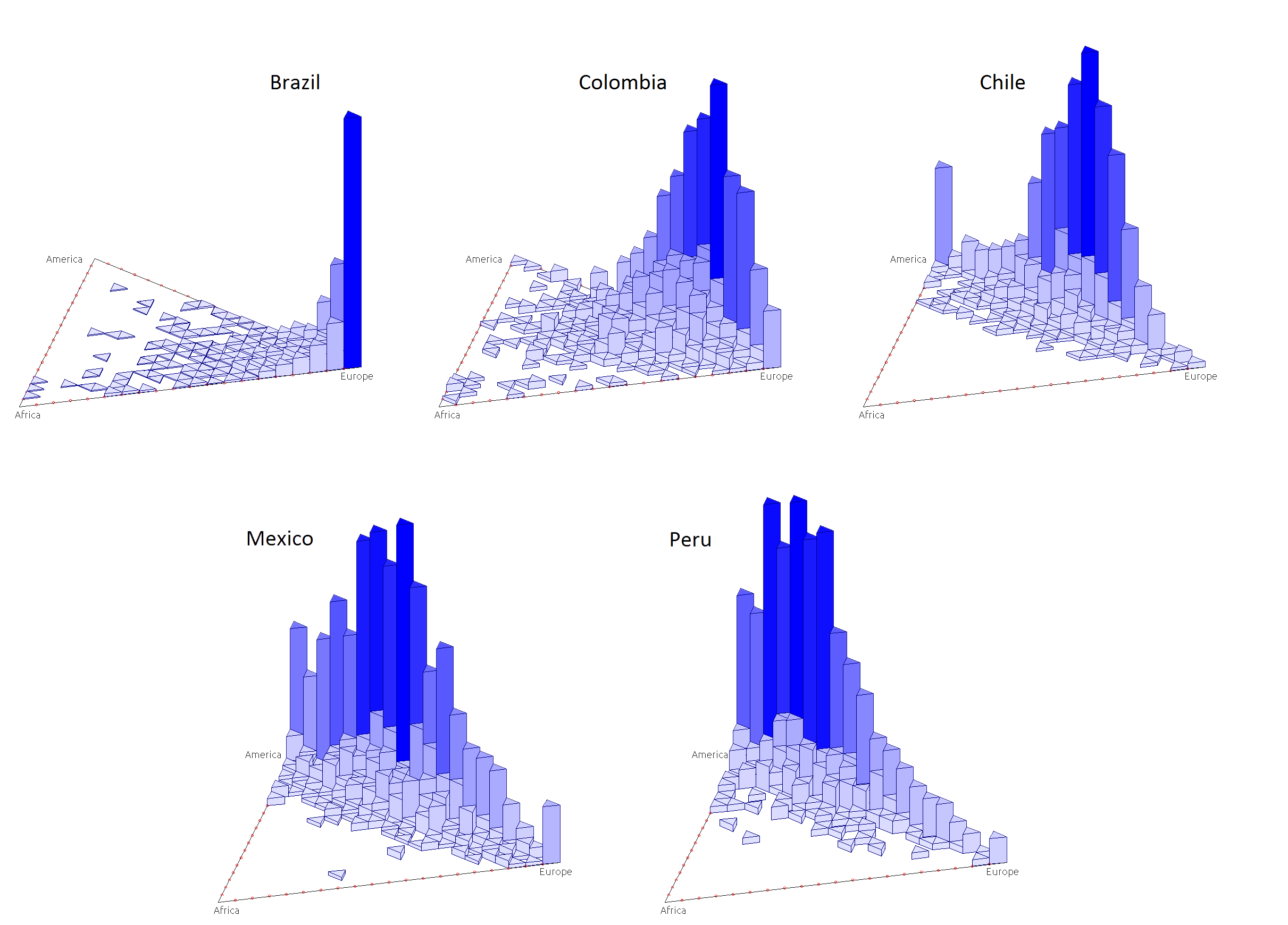


The sample collected in Brazil has a highly predominant European ancestry (median 82%) and relatively low Native American and African ancestries (both medians ~9%) (Table 1). The samples collected in Chile, México and Perú have the highest median Native American ancestry (48%, 56% and 64% respectively) and lowest median African ancestry (5%, 5% and 0% respectively). The sample collected in Colombia is the most diverse in showing a predominantly European ancestry (median 60%) but substantial Native American admixture (median 29%) and the highest African component (median 11%). There is considerable heterogeneity across ascertainment sites in the distribution of individual ancestry estimates (Figures below). The Mexican sample is characterized by the widest spread of individual ancestry along the Native American-European axis. By contrast the Chilean sample, shows a greater concentration around the median. The Brazilian, and particularly the Colombian samples, show a greater spread of ancestry along the African axis, with some individuals having high (>70%) estimated African ancestry.
