# Supplementary material for: Admixture in Latin America: Geographic Structure, Phenotypic Diversity and Self-Perception of Ancestry Based on 7,342 Individuals
Source: PLoS Genet. 2014 Sep 25;10(9):e1004572. doi: 10.1371/journal.pgen.1004572 (PMC4177621; doi:10.1371/journal.pgen.1004572)

## Supplementary Text S2: Correlation of socioeconomic position with ancestry

European ancestry increases with higher educational attainment and wealth. The box-plots below illustrate this trend in the global sample, for education (recorded in three levels: 1-None/primary/technical 2-Secondary 3-University and post-graduate) and for wealth (in deciles of the wealth index). The trend is highly significant (r= 0.12, p-value <2.2×10^-16^ for both socioeconomic indicators). Similar results are obtained in each country (not shown).


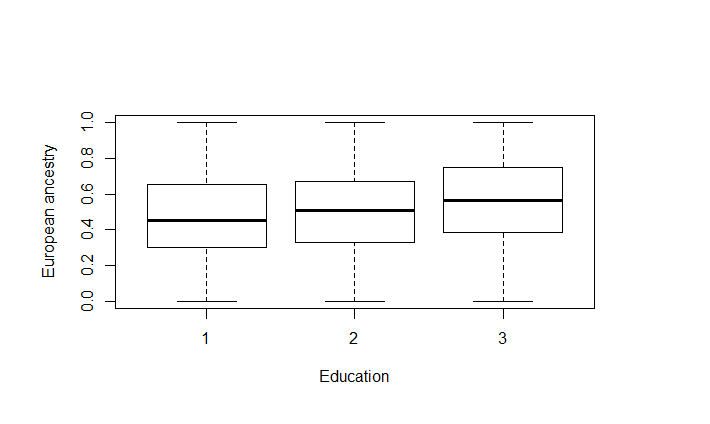


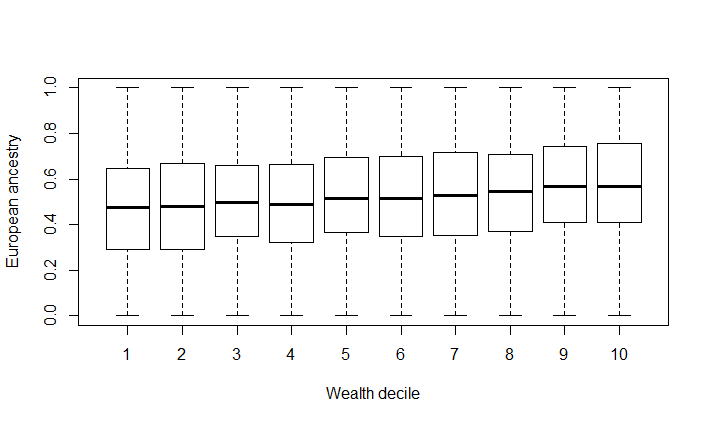

Supplement: Text S2 — Correlation of socioeconomic position with ancestry. (DOCX) [file pgen.1004572.s012.docx]
