# Supplementary material for: Admixture in Latin America: Geographic Structure, Phenotypic Diversity and Self-Perception of Ancestry Based on 7,342 Individuals
Source: PLoS Genet. 2014 Sep 25;10(9):e1004572. doi: 10.1371/journal.pgen.1004572 (PMC4177621; doi:10.1371/journal.pgen.1004572)
Supplement: Text S3 — Regression of height (in cm) on ancestry and covariates. (DOCX) [file pgen.1004572.s013.docx]

## Supplementary Text S3: Regression of height (in cm) on ancestry and covariates.

|  | Coefficient | p-value |
| --- | --- | --- |
| European Ancestry | 7.308 | <2.00E-16 |
| African Ancestry | 8.136 | <2.00E-16 |
| Age | -0.093 | <2.00E-16 |
| Sex-Male | 13.084 | <2.00E-16 |
| Country-Chile | -0.998 | 6.60E-04 |
| Country-Colombia | -1.434 | 4.13E-09 |
| Country-Mexico | -0.284 | 3.65E-01 |
| Country-Peru | -0.701 | 6.32E-02 |
| Education | 0.781 | 8.20E-12 |
| Wealth | 0.089 | 6.89E-04 |

Multiple R-squared: 0.55
