# Supplementary material for: Admixture in Latin America: Geographic Structure, Phenotypic Diversity and Self-Perception of Ancestry Based on 7,342 Individuals
Source: PLoS Genet. 2014 Sep 25;10(9):e1004572. doi: 10.1371/journal.pgen.1004572 (PMC4177621; doi:10.1371/journal.pgen.1004572)
Supplement: Text S4 — Regression of hair shape on marker rs260690 and covariates. (DOCX) [file pgen.1004572.s014.docx]

## Supplementary Text S4. Regression of hair shape on genotype at marker rs260690 and covariates.

|  | Coefficient | p-value |
| --- | --- | --- |
| rs260690 | -0.192 | <2.00E-16 |
| European Ancestry | 0.099 | 1.61E-01 |
| African Ancestry | 2.318 | <2.00E-16 |
| Age | -0.001 | 2.29E-01 |
| Sex-Male | 0.017 | 4.13E-01 |
| Country-Chile | -0.084 | 3.87E-02 |
| Country-Colombia | 0.145 | 8.92E-06 |
| Country-Mexico | 0.161 | 1.25E-04 |
| Country-Peru | 0.290 | 1.01E-03 |
| Education | -0.006 | 6.30E-01 |
| Wealth | -0.004 | 5.45E-01 |

Multiple R-squared: 0.15

Marker rs260690 is located in the first intron of the EDAR gene. Examination of HapMap data for Mexican Americans (MEX) indicates high linkage disequilibrium (*D*^’^=0. 98) between this SNP and rs3827760, an A to G variant coding for a p.Val370Ala substitution in EDAR. The derived G allele at rs3827760 has a high frequency in CHB+JPT (0.95) but is rare in CEU+TSI (frequency 0.05) and this allele has recently been associated with increased cross-sectional hair thickness in East Asians as well as in mouse models[[1-4](#_ENREF_1)].

1. Fujimoto A, Kimura R, Ohashi J, Omi K, Yuliwulandari R, et al. (2008) A scan for genetic determinants of human hair morphology: EDAR is associated with Asian hair thickness. Hum Mol Genet 17: 835-843.

2. Fujimoto A, Ohashi J, Nishida N, Miyagawa T, Morishita Y, et al. (2008) A replication study confirmed the EDAR gene to be a major contributor to population differentiation regarding head hair thickness in Asia. Hum Genet 124: 179-185.

3. Mou C, Thomason HA, Willan PM, Clowes C, Harris WE, et al. (2008) Enhanced ectodysplasin-A receptor (EDAR) signaling alters multiple fiber characteristics to produce the East Asian hair form. Hum Mutat 29: 1405-1411.

4. Tan J, Yang Y, Tang K, Sabeti PC, Jin L, et al. (2013) The adaptive variant EDARV370A is associated with straight hair in East Asians. Hum Genet 132: 1187-1191.
