# Supplementary material for: Admixture in Latin America: Geographic Structure, Phenotypic Diversity and Self-Perception of Ancestry Based on 7,342 Individuals
Source: PLoS Genet. 2014 Sep 25;10(9):e1004572. doi: 10.1371/journal.pgen.1004572 (PMC4177621; doi:10.1371/journal.pgen.1004572)
Supplement: Text S5 — Description of face shape changes associated with PCs 1–5 of 3D landmark coordinates. (DOCX) [file pgen.1004572.s015.docx]

## Supplementary Text S5. Description of face shape changes associated with PCs 1-5 of 3D landmark coordinates.

These PCs are shown in Supplementary Figure S4

PC1

The first PC explains changes on the overall shape of the face, with shorter, narrower and anteriorly projected faces occupying the positive values of the PC axis. Also, positive scores across the first PC are characterized by shorter noses, eyes displaced downwards and lips displaced upwards.

PC2

Positive values across the second PC correspond to shorter and wider faces, depicting also a more superior position of the lips, and a lower localization of the eyes. Anterior projection of the chin is characteristic of individuals occupying the positive values, whereas the nose and the glabellar region are more projected on the negative values of the second PC axis.

PC3

PC3 is mainly dominated by facial flatness, with more flattened faces on the negative values. Facial flatness can be seen as composite changes including the anterior displacement of the eyes and frontal region, along with a posterior movement of lips, chin and the inferior face in general. Negative values across this PC (flattened faces) also present more lateralized and downwardly displaced earlobes.

PC4

PC4 can be seen as a descriptor of changes occurring in the superior face, more specifically in the position of the frontotemporale. Individuals occupying the negative scores of this PC present marked glabellar and nasal projection accompanied by a downward displacement of the frontotemporal relative to the eyes. In contrast, the superior lips are elevated towards the inferior part of the nose on individuals placed at the positive values.

PC5

The fifth PC describes variations attaining the whole configuration of landmarks, and involving a downward displacement and narrowing of the nose, a shortening of the lips, and a more superior position of the superciliares in the positive values. Conversely, negative scores are depicted by more laterally placed eyes, more medially placed earlobes, and wider frontals.
