# Supplementary material for: Admixture in Latin America: Geographic Structure, Phenotypic Diversity and Self-Perception of Ancestry Based on 7,342 Individuals
Source: PLoS Genet. 2014 Sep 25;10(9):e1004572. doi: 10.1371/journal.pgen.1004572 (PMC4177621; doi:10.1371/journal.pgen.1004572)
Supplement: Text S6 — Obtaining population size at individual birthplaces. (DOCX) [file pgen.1004572.s016.docx]

## Supplementary Text S6. Obtaining population size at individual birthplaces.

To obtain census size at each sampled individual birthplace, we used the Global Rural-Urban Mapping Project^1^ (GRUMP), version 1. This is the most informative census size data set for Latin American locations. Because the birth location name was given by the volunteers themselves, sometimes that location name differs from the one in GRUMP. Also, the geographic coordinates of birth locations obtained from GeodesiX differs slightly from the ones in GRUMP. Due to this we performed an automatic assignment of each birth place in our database to its geographically closest settlement location in the GRUMP database. To achieve this we used the *Near Table* and *Table Join* functions in ArcGIS 9.3.1 (ESRI, Redlands).

For each location, GRUMP includes census sizes for the years 2000, 1995, and 1990. We chose to use 1990 as it is likely to be closer in time to the birth dates of sampled individuals, the median age in our dataset being in the range of 20-25.

^1^Center for International Earth Science Information Network (CIESIN), Columbia University; International Food Policy Research Institute (IFPRI), the World Bank; and Centro Internacional de Agricultura Tropical (CIAT). 2011.

Global Rural-Urban Mapping Project, Version 1 (GRUMPv1): Settlement Points. Palisades, NY: Socioeconomic Data and Applications Center (SEDAC), Columbia University. Available at http://sedac.ciesin.columbia.edu/data/dataset/grump-v1-settlement-points (Downloaded April 7, 2014)
